# FGFR4 Inhibitor BLU9931 Attenuates Pancreatic Cancer Cell Proliferation and Invasion While Inducing Senescence: Evidence for Senolytic Therapy Potential in Pancreatic Cancer

**DOI:** 10.3390/cancers12102976

**Published:** 2020-10-14

**Authors:** Norihiko Sasaki, Fujiya Gomi, Hisashi Yoshimura, Masami Yamamoto, Yoko Matsuda, Masaki Michishita, Hitoshi Hatakeyama, Yoichi Kawano, Masashi Toyoda, Murray Korc, Toshiyuki Ishiwata

**Affiliations:** 1 Research team for Geriatric Medicine (Vascular Medicine), Tokyo Metropolitan Institute of Gerontology, Sakae-cho 35-2, Itabashi-ku, Tokyo 173-0015, Japan; sasanori@tmig.or.jp (N.S.); mtoyoda@tmig.or.jp (M.T.); 2 Division of Aging and Carcinogenesis, Research Team for Geriatric Pathology, Tokyo Metropolitan Institute of Gerontology, Tokyo 173-0015, Japan; gomif@tmig.or.jp; 3 Division of Physiological Pathology, Department of Applied Science, School of Veterinary Nursing and Technology, Nippon Veterinary and Life Science University, Tokyo 180-8602, Japan; yoshimura-h@nvlu.ac.jp (H.Y.); masami@nvlu.ac.jp (M.Y.); 4 Oncology Pathology, Department of Pathology and Host-Defense, Kagawa University, Kagawa 761-0793, Japan; youkoh@med.kagawa-u.ac.jp; 5 Department of Veterinary Pathology, School of Veterinary Medicine, Nippon Veterinary and Life Science University, Tokyo 180-8602, Japan; michishita@nvlu.ac.jp; 6 Department of Comprehensive Education in Veterinary Medicine, Nippon Veterinary and Life Science University, Tokyo 180-8602, Japan; hatakeyama@nvlu.ac.jp; 7 Department of Gastrointestinal and Hepato-Biliary-Pancreatic Surgery, Nippon Medical School, Tokyo 113-8603, Japan; y-kawano@nms.ac.jp; 8 Department of Developmental and Cell Biology, School of Biological Sciences, University of California, Irvine, CA 92697, USA; mkorc@uci.edu

**Keywords:** FGFR4, pancreatic cancer, FGFR4 inhibitor, growth, invasion, senescence, senolytic therapy

## Abstract

**Simple Summary:**

Pancreatic ductal adenocarcinoma (PDAC) is a deadly malignancy that is projected to become the leading cause of cancer death by 2050. Fibroblast growth factor receptor 4 (FGFR4) is a transmembrane receptor that is overexpressed in half of PDACs. We determined that its expression in PDAC positively correlated with larger tumor size and more advanced tumor stage, and that BLU9931, a selective FGFR4 inhibitor, reduced PDAC cell proliferation and invasion while promoting their senescence. Quercetin, a senolytic drug, induced cell death in BLU9931-treated cells. We propose that targeting FGFR4 in combination with senolysis could provide a novel therapeutic strategy in patients whose PDAC expresses high FGFR4 levels.

**Abstract:**

Fibroblast growth factor receptor 4 (FGFR4), one of four tyrosine kinase receptors for FGFs, is involved in diverse cellular processes. Activation of FGF19/FGFR4 signaling is closely associated with cancer development and progression. In this study, we examined the expression and roles of FGF19/FGFR4 signaling in human pancreatic ductal adenocarcinoma (PDAC). In human PDAC cases, FGFR4 expression positively correlated with larger primary tumors and more advanced stages. Among eight PDAC cell lines, FGFR4 was expressed at the highest levels in PK-1 cells, in which single-nucleotide polymorphism G388R in *FGFR4* was detected. For inhibition of autocrine/paracrine FGF19/FGFR4 signaling, we used BLU9931, a highly selective FGFR4 inhibitor. Inhibition of signal transduction through ERK, AKT, and STAT3 pathways by BLU9931 reduced proliferation in FGF19/FGFR4 signaling-activated PDAC cells. By contrast, BLU9931 did not alter stemness features, including stemness marker expression, anticancer drug resistance, and sphere-forming ability. However, BLU9931 inhibited cell invasion, in part, by downregulating membrane-type matrix metalloproteinase-1 in FGF19/FGFR4 signaling-activated PDAC cells. Furthermore, downregulation of SIRT1 and SIRT6 by BLU9931 contributed to senescence induction, priming these cells for quercetin-induced death, a process termed senolysis. Thus, we propose that BLU9931 is a promising therapeutic agent in FGFR4-positive PDAC, especially when combined with senolysis (195/200).

## 1. Introduction

Pancreatic ductal adenocarcinoma (PDAC) is a deadly cancer with a 5-year survival rate of ~9% [1]. PDAC is the third leading cause of cancer death in the USA, and the fourth leading cause of cancer death in Japan [2]. PDAC is projected to become the leading cause of cancer death by 2050 [3]. Surgery offers the only hope for a possible cure for PDAC. However, 80% of PDAC patients are inoperable at diagnosis. Despite the development of non-surgical therapies, including chemotherapy, radiotherapy, and chemoradiotherapy, many of these patients die less than 6 months following diagnosis. Even in patients who undergo resection, recurrence and development of distant metastasis occur frequently [4]. Fibroblast growth factors (FGFs) constitute a large family of growth factors, including FGF1 through FGF23 [5]. FGFs exert their biological activities by binding to high-affinity tyrosine kinase FGF receptors (FGFRs) on the surface of cells and low-affinity heparan sulfate proteoglycans that enhance ligand presentation [6,7]. Most FGFs are heparin binding, but FGF19, FGF21, and FGF23 do not have a heparin-binding domain and exhibit a low affinity for heparan sulfates [8]. These three FGFs are viewed as hormones rather than growth factors since they are readily released into the systemic circulation and modulate metabolic functions [8]. 

FGFRs are transmembrane receptors, each consisting of an extracellular, transmembrane, and intracellular domain. The extracellular domains of FGFRs are usually composed of three immunoglobulin-like regions (Ig I–III), whereas their intracellular tyrosine kinase site is discontinuous due to the presence of a non-kinase intervening sequence [5,6,9]. There are four FGFR family members, named FGFR1, 2, 3, and 4, each of which is encoded by a distinct gene [5,6,9]. The C-terminal halves of the third Ig-like domains of FGFR1-3 undergo alternative splicing to generate the respective IIIb and IIIc variants, whereas FGFR4 does not possess such alternative exons [5,6,7,8,9,10]. Expression of FGFRs and their corresponding ligands (FGFs) contributes to tumor progression in human malignancies by enhancing proliferation, invasion, and angiogenesis via both autocrine and paracrine effects [11,12,13,14,15,16,17]. FGF19 requires the presence of β-Klotho to efficiently bind and activate FGFR4 [18]. By contrast, FGF21 and FGF23 signal mostly through FGFR1c and FGFR2c, but FGF21 requires β-Klotho whereas FGF23 requires α-Klotho for efficient binding to these FGFRs [18,19].

FGFR4 is reportedly overexpressed in various cancers, such as breast, prostate, hepatocellular, ovarian, gastric, colorectal, and pancreatic cancer, where it may contribute to tumor progression [20,21,22,23]. Inhibition of FGFR4 suppresses the aggressiveness of gastric, colorectal, and serous ovarian cancers that are high grade [21,24,25]. In the pancreas, FGFR4 expression is markedly increased in advanced pancreatic intra-epithelial neoplasia (PanIN) and PDAC compared with the normal pancreas and low-grade PanIN [23]. Moreover, a single-nucleotide polymorphism (SNP) in exon 9 of the gene encoding FGFR4 results in the substitution of glycine for arginine at codon 388 (388 Gly/Arg) in the transmembrane domain and is associated with poor outcome in high-grade soft tissue sarcoma, lung, and breast cancers [26,27,28]. In pancreatic neuroendocrine tumors (PNETs), transfection of FGFR4-G388R vector promoted tumor progression by increasing intraperitoneal spread and liver metastasis in immunodeficient mice [29]. FGF19/FGFR4 signaling is implicated in many cellular processes, such as cell proliferation, migration, metabolism, and differentiation [30,31]. Furthermore, activation of FGF19/FGFR4 signaling is closely associated with cancer development and progression [32,33]. Therefore, FGF19/FGFR4 signaling pathways could be attractive targets for anticancer therapeutics [34,35]. 

BLU9931 is an irreversible and highly selective small molecule inhibitor for FGFR4 [35]. BLU9931 makes a covalent bond with Cys552 within the ATP-binding pocket of FGFR4, which is not present in FGFR1-3. BLU9931 inhibited gastric cancer cell growth in a transplanted mouse model [36]. To date, however, there have been no reports about the effects of FGFR4 inhibitors in PDAC. In the present study, therefore, we delineated FGFR4 expression in PDAC, and examined the actions of BLU9931 on FGF19/FGFR4 signaling and biological processes in PDAC cells.

## 2. Results

### 2.1. Expression of FGFR4 in Human PDAC Tissues and PDAC Cell Lines

**Figure 1:**
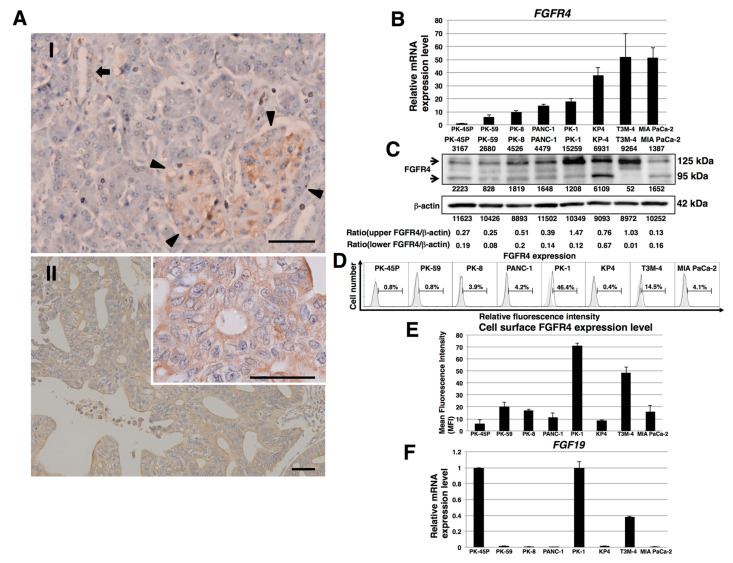
FGFR4 expression in human pancreatic tissues and PDAC cell lines. (**A**) Representative photographs of immunohistochemistry for FGFR4. In normal human pancreatic tissues, weak FGFR4 immunoreactivity was present in the normal exocrine and endocrine pancreas. AI: Arrowheads indicate endocrine islets, whereas arrow indicates ductal cells. AII: Strong FGFR4 immunoreactivity was present in the cytoplasm and cell membrane of human PDAC cells. Scale bar: 200 μm; *n* = 136 PDAC cases. Inset: strong membranous FGFR4 immunoreactivity is readily evident. Scale bar: 200 μm (**B**) Real-time qPCR analysis of *FGFR4* in PDAC cell lines. Representative results from triplicate measurements are shown. Results shown were normalized to values obtained for PK-45P cells (value = 1). (**C**) Western blot analysis of FGFR4 was performed in PDAC cell lines. The expression of each band is shown under or above the blot. (**D**) FACS analysis of FGFR4 expression in PDAC cell lines. Controls are indicated by thin lines with gray color. (**E**) Cell surface levels of FGFR4 expression in PDAC cell lines. Mean fluorescence intensities (MFIs) from FACS analysis are shown. Results are presented as means ± SD from three independent experiments. (**F**) Real-time qPCR analysis of *FGF19* in PDAC cell lines. Representative results from triplicate measurements are shown. Control PK-45P cells were assigned a value of 1, and all other values in this series of experiments were calculated in relation to this reference control value. (value = 1).

To examine the expression of FGFR4 in human PDAC tissues, we performed immunohistochemical analysis of FGFR4 using pancreas tissue microarrays. In normal human pancreatic tissues, FGFR4 was weakly localized in normal exocrine and endocrine pancreatic tissues (Figure 1(A-I)). FGFR4 was observed in the cytoplasm and cell membrane of human PDAC cells (Figure 1(A-II) and Figure S1). Strong FGFR4 immunoreactivity was seen in the cancer cells in 67 of 136 (49%) PDAC samples and high FGFR4 expression correlated with larger primary tumor size and more advanced stages of the cancer (*p* < 0.001, Table 1).

*FGFR4* mRNA was expressed in all eight PDAC cell lines examined (Figure 1B). *FGFR4* mRNA was highest in T3M-4 and MIA PaCa-2 cells and lowest in PK-45P cells. By contrast, FGFR4 protein levels were highest in PK-1 and T3M-4 cells and lowest in MIA PaCa-2 and PK-45P cells (Figure 1C). Fluorescence-activated cell sorting revealed that cell surface FGFR4 levels were highest in PK-1 and T3M-4 cells (Figure 1D,E). Given that SNP Arg388 of the *FGFR4* gene may be associated with decreased survival in certain cancers, we next examined this SNP in the above cell lines, which genotyped as follows: MIA PaCa-2 and PK-8 cells as Gly/Gly; PK-1, PANC-1, and PK-45P cells as Gly/Arg; and PK-59, T3M-4, and KP4 cells as Arg/Arg (Figure S2 and Table 2). Inasmuch as FGF19 is the specific and sole ligand for FGFR4, we next performed real-time quantitative PCR (qPCR) for this ligand. *FGF19* mRNA levels were relatively elevated in PK-1, PK-45P, and T3M-4 cells, and lowest in other cell lines (Figure 1F). 

**Figure 2:**
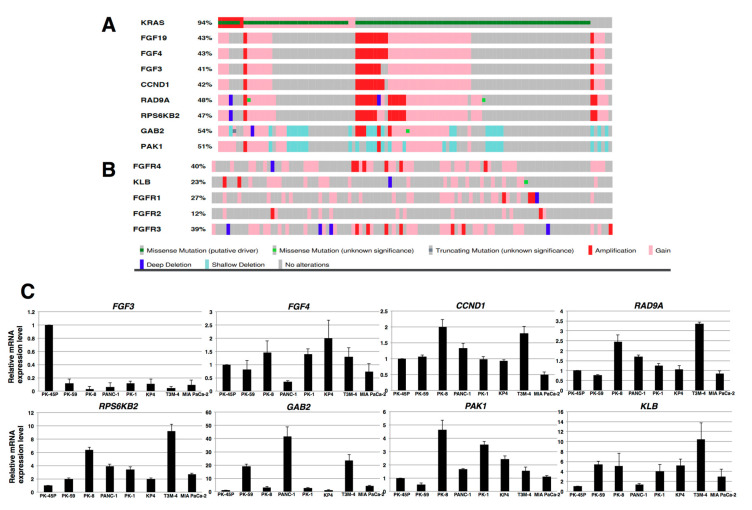
Oncoprints visualizing the *FGF19* amplicon and analysis of related genes in human PDAC. (**A**) TCGA analysis of the 11q13.3 amplicon in PDAC revealed the presence of the following amplified genes: *FGF19*, *FGF4*, *FGF3*, *CCND1*, *RAD9A*, *RPS6KB2*, *GAB2*, and to a lesser extent *PAK1*. (**B**) TCGA data for *FGFR4* encoding the receptor that is activated by FGF19, *KLB* encoding β-klotho, and *FGFR1*, *FGFR2*, and *FGFR3* genes. (**C**) Real-time qPCR analysis of RNA from indicated cell lines was performed for *FGF4*, *FGF3*, *CCND1*, *RAD9A*, *RPS6KB2*, *GAB2*, *PAK1*, and *KLB*. Representative results from triplicate measurements are shown. Results shown were normalized to values obtained for PK-45P cells (value = 1).

The *FGF19* gene is occasionally amplified in hepatocellular carcinomas and breast cancer as a consequence of the presence of an amplicon on chromosome 11q13.3 [37,38]. PDAC also exhibits regions of genomic amplification, including the 11q13.3 region in chromosome 11 [39]. To determine whether such an amplicon harbors an *FGF19* amplification, we examined gene expression data in The Cancer Genome Atlas (TCGA), a publicly available data base [40,41]. We used the University of Texas Southwestern (UTSW) data set since it was based on high-quality cancer cell-enriched samples [42], which revealed the presence of an amplified *FGF19* gene in 11 of the 109 samples in this cohort (Figure 2A). In addition, other genes within this amplicon were also amplified, including *FGF4*, *FGF3*, *CCND1*, *RAD9A*, *RPS6KB2*, *GAB2*, and *PAK1* (Figure 2A). In some cases, there was also co-amplification of *FGF19* with *FGFR4* (Figure 2B), even though the *FGFR4* gene is located on chromosome 5q25. Only 7 of the 109 samples exhibited concomitant upregulation of the *KLB* gene (encoding β-Klotho) and *FGFR4* gene (Figure 2B). Only three and two cases exhibited *FGFR1* and *FGFR2* gene amplification, respectively, whereas seven cases exhibited *FGFR3* amplification, including one case that did not have a *KRAS* mutation. In three patients, there was concomitant overexpression of *FGFR3* and *FGFR4*. Intriguingly, among the eight PDAC cases that did not harbor a *KRAS* mutation, four cases had either amplification or increased expression of the *FGF19*, *FGF4*, *FGF3*, and *CCND1* genes, and six cases had *RAD9A* and *RPS6KB2* gene overexpression, two of which were due to amplification (Figure 2A), raising the possibility that a portion of the 11q13.3 amplicon helps drive PDAC development in the absence of mutated *KRAS*. Additional real-time qPCR analysis of RNA from the above cell lines for *FGF4*, *FGF3*, *CCND1*, *RAD9A*, *RPS6KB2*, *GAB2*, *PAK1*, and *KLB* revealed variable increases in these mRNA moieties in these cells, most notably with respect to *RPS6KB2*, *GAB2*, *PAK1*, and *KLB* (Figure 2C). Taken together, these results suggest that autocrine/paracrine FGF19/FGFR4 signaling may be functional in PK-1 and T3M-4 cells. Therefore, most subsequent experiments with cell lines were performed using PK-1 and T3M-4 cell lines.

### 2.2. Effects of BLU9931 on Growth and Cell Cycle of PDAC Cells

**Figure 3:**
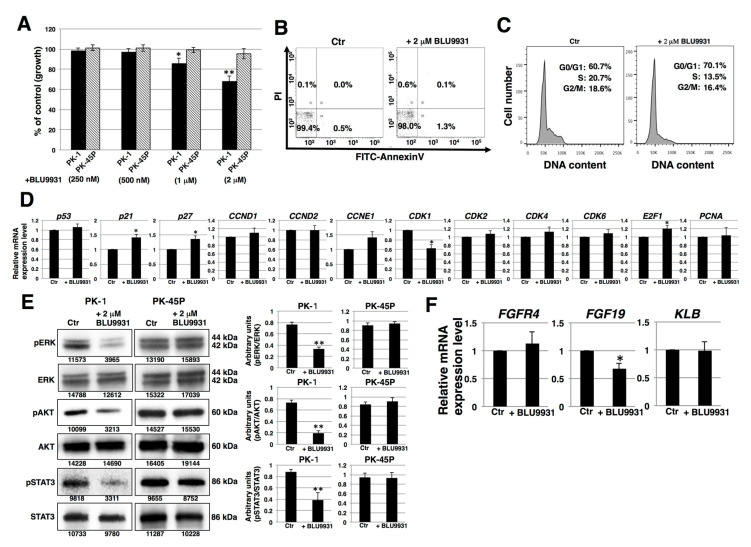
Effects of BLU9931 on PDAC cells. (**A**) PDAC cells were incubated with or without BLU9931 for 3 days at the indicated concentrations and growth rates were determined. Results are presented as means ± SD from three independent experiments. (**B**) Quantification of apoptotic, necrotic, and live cells by flow cytometry in PK-1 cells incubated with or without 2 μM BLU9931 for 3 days. (**C**) Cell cycle analysis in PK-1 cells that were incubated with or without 2 μM BLU9931 for 3 days. (**D**) Real-time qPCR analysis of cell cycle-related genes in PK-1 cells that were incubated with or without 2 μM BLU9931 for 3 days. Results shown are normalized to values obtained for control cells (value = 1). Results are presented as means ± SD from three independent experiments. (**E**) Western blot analysis for FGF19/FGFR4 signaling was performed in PK-1 or PK-45P cells that were incubated with or without 2 μM BLU9931 for 3 days. The expression of each band is shown under the blot. The histograms show mean densitometric readings ± SD for the phosphorylated proteins normalized to those of the loading controls. Results are the means ± SD from three independent experiments. (**F**) Real-time qPCR analysis of *FGFR4* or *FGF19* or *KLB* in PK-1 cells that were incubated with or without 2 μM BLU9931 for 3 days. Results are normalized to values obtained for control cells (value = 1), and are the as means ± SD from three independent experiments. * *p* < 0.05, ** *p* < 0.01. Control (Ctr): Control cells were incubated with dimethyl sulfoxide.

To determine whether there was a functional FGF19/FGFR4 signaling axis in human PDAC cells, we cultured PDAC cells with or without the highly specific FGFR4 inhibitor, BLU9931. PK-1 cells exhibited a dose-dependent decrease in proliferation in the presence of BLU9931 (Figure 3A). An inhibitory effect on proliferation was also observed in T3M-4 cells (Figure S3A). By contrast, BLU9931 did no inhibit proliferation in PK-45P cells (Figure 3A), which expressed very low FGFR4 levels (Figure 1). Next, we examined whether BLU9931 exerts toxic effects in PDAC cells. Incubation of PK-1 cells with 2 μM BLU9931 followed by fluorescence activated cell sorter (FACS) analysis using annexin V and propidium iodide (PI) showed that ~2% of the cells underwent apoptosis, necrosis, or cell injury (Figure 3B). Higher concentration (>10 μM) of BLU9931 revealed dead and floating cells suggestive of a toxic effect at these very high concentrations of the drug. 

We next examined the effects of BLU9931 on the cell cycle by FACS analysis. There was a higher percentage of BLU9931-treated PK-1 or T3M-4 cells in the G0/G1 phase (70.1% vs. 60.7% and 66.4% vs. 58.0%, respectively) and lower percentage in the S phase (13.5% vs. 20.7% and 27.3% vs. 29.9%, respectively) and G2/M phase (16.4% vs. 18.6% and 6.3% vs. 12.1%, respectively) relative to control cells (Figure 3C and Figure S3B). These results point to a G1 to S phase cell cycle slowdown in BLU9931-treated cells.

Next, we examined the expression of cell cycle related-genes. Real-time qPCR analysis showed that the expression of *p21*, *P27*, and *E2F1* was upregulated whereas *CDK1* expression was downregulated in BLU9931-treated cells (Figure 3D and Figure S3C). We also examined autocrine/paracrine FGF19/FGFR4 signaling by immunoblotting for downstream targets (ERK, AKT, and STAT3). As shown in Figure 3E, reduced phosphorylation of ERK, AKT, and STAT3 was observed in BLU9931-treated PK-1 cells. Similar reductions in phosphorylation in these downstream signaling molecules were observed in T3M-4 cells (Figure S3D). By contrast, phosphorylation of these proteins was not altered by the same concentration of BLU9931 (2 μM) in PK-45P cells. These results indicate that autocrine/paracrine FGF19/FGFR4 signaling is functional in PK-1 and T3M-4 cells but not in PK-45P cells, and that FGFR4 downstream signaling can be inhibited by BLU9931 in certain PDAC cells. Furthermore, BLU9931 reduced *FGF19* mRNA levels but not *FGFR4* and *KLB* mRNA levels (Figure 3F and Figure S3E), suggesting that autocrine/paracrine FGF19/FGFR4 signaling upregulates FGF19 expression through the FGF19/FGFR4 signaling feedback loop. Taken together, these results indicate that the inhibitory effect of 2 μM BLU9931 on PDAC cell proliferation is caused by inhibition of autocrine/paracrine FGF19/FGFR4 signaling and not through induction of apoptosis.

### 2.3. Effects of BLU9931 on Stemness Features of PDAC Cells

**Figure 4:**
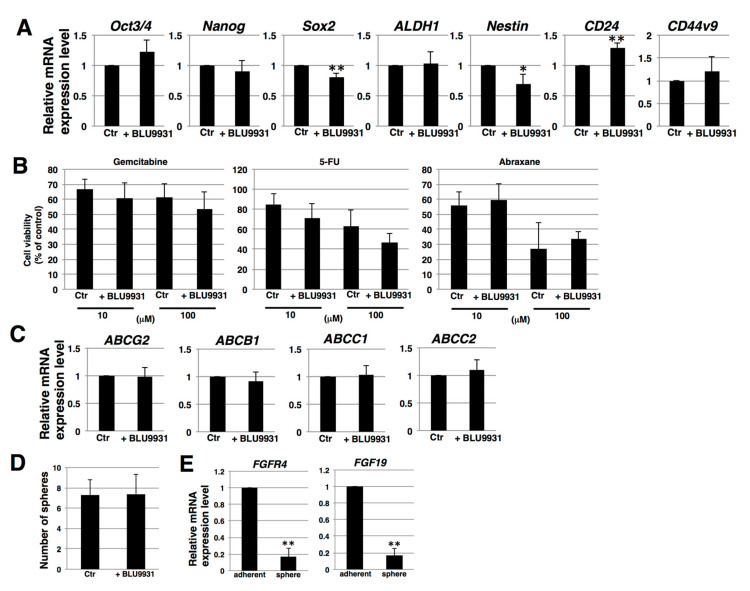
Effects of BLU9931 on stemness features of PDAC cells. (**A**) Real-time qPCR analysis of stemness markers in PK-1 cells that were incubated with or without 2 μM BLU9931 for 3 days. Results shown are normalized to values obtained for control cells (value = 1). (**B**) Anticancer drug resistance assay in PK-1 cells that were incubated with or without 2 μM BLU9931. The dose response (10 or 100 μM) of PK-1 cells to gemcitabine, 5-FU, and abraxane was determined using the ATP assay. (**C**) Real-time qPCR analysis of transporters in PK-1 cells that were incubated with or without 2 μM BLU9931 for 3 days. Results shown are normalized to values obtained for control cells (value = 1). (**D**) Sphere-forming assays performed in PK-1 cells that were incubated with or without 2 μM BLU9931. (**E**) Real-time qPCR analysis of *FGFR4* or *FGF19* in PK-1 cells cultured under adherent or 3-D conditions. Results shown are normalized to values obtained for adherent-cultured cells (value = 1). All histograms are presented as means ± SD from three independent experiments. * *p* < 0.05, ** *p* < 0.01. Control (Ctr): Control cells were incubated with dimethyl sulfoxide.

To clarify the correlation between autocrine/paracrine FGF19/FGFR4 signaling and stemness in PDAC cells, we examined stemness features, including stemness marker expression, anticancer drug resistance, and sphere-forming ability. Real-time qPCR showed that two of seven examined stemness markers (*Sox2* and *Nestin*) were expressed at lower levels in 2 μM BLU9931-treated PK-1 cells (Figure 4A), whereas one of the seven markers (*CD24*) was expressed at a higher level in BLU9931-treated PK-1 than in control cells (Figure 4A). These results suggest that autocrine/paracrine FGF19/FGFR4 signaling is not definitively involved in stemness marker expression in PDAC cells. To assess anticancer drug resistance, we used three anti-pancreatic cancer drugs, gemcitabine, 5-FU, and abraxane. Cell survival rates following addition of gemcitabine, 5-FU, and abraxane (all at 100 μM) were approximately 60%, 50%, and 30%, respectively (Figure 4B). However, survival rates were not significantly different in 2 μM BLU9931-treated PK-1 cells following incubation with the above anticancer drugs at either 10 or 100 μM (Figure 4B). Furthermore, we examined the expression levels of four potential anticancer drug transporters. Real-time qPCR analysis revealed that the expression of *ABCG2*, *ABCB1*, *ABCC1*, and *ABCC2* was not significantly different between control and 2 μM BLU9931-treated PK-1 cells (Figure 4C). Thus, autocrine/paracrine FGF19/FGFR4 signaling is not involved in regulating the expression of anticancer drug transporters and the resistance toward anticancer drugs in PDAC cells. Next, we examined sphere-forming ability, which is viewed as an indicator of self-renewal and stemness [43,44,45]. Sphere-forming assays showed that the number of spheres was not different between control and 2 μM BLU9931-treated PK-1 cells (Figure 4D). Furthermore, in sphere cells, the expression levels of *FGFR4* and *FGF19* were markedly lower than those in adherent cells (Figure 4E). Thus, autocrine/paracrine FGF19/FGFR4 signaling is not involved in sphere formation of PDAC cells.

### 2.4. Effects of BLU9931 on Migration and Invasion of PDAC Cells

**Figure 5:**
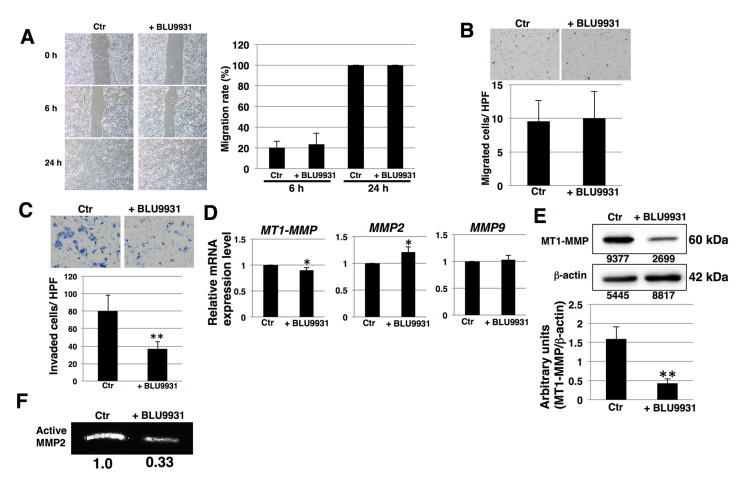
Effects of BLU9931 on migration and invasion of PDAC cells. (**A**) Wound healing/cell scratch assay was performed in PK-1 cells incubated with or without 2 μM BLU9931 for 3 days. Images show the times (0, 6, and 24 h) after the ibidi 2-well culture-insert was removed. The histograms show the average normalization of the wound migration rate in three experiments. (**B**) Migration assays were performed in PK-1 cells incubated with or without 2 μM BLU9931 for 3 days. Representative results from measurements of 12 fields are shown. (**C**) Matrigel invasion assays performed in PK-1 cells incubated with or without 2 μM BLU9931 for 3 days. Representative results from measurements of 12 fields are shown. (**D**) Real-time qPCR analysis of *MMPs* in PK-1 cells incubated with or without 2 μM BLU9931 for 3 days. Results shown are normalized to values obtained for control cells (value = 1). Results are the means ± SD from three independent experiments. (**E**) Western blot analysis of MT1-MMP was performed in PK-1 that were incubated with or without 2 μM BLU9931 for 3 days. The expression of each band is shown under the blot. The histograms show mean densitometric readings ± SD for MT1-MMP normalized to those of the loading controls. Results are the as means ± SD from three independent experiments. (**F**) Gelatin zymography was performed using culture supernatants from PK-1 cells that were incubated with or without 2 μM BLU9931 for 3 days. Relative band intensity is shown. * *p* < 0.05, ** *p* < 0.01. Control (Ctr): Control cells were incubated with dimethyl sulfoxide.

We next examined the inhibitory effects of FGF19/FGFR4 signaling on migration and invasion of PDAC cells. In wound healing assays, there were no significant differences in the migration rates between control and BLU9931-treated PK-1 cells (Figure 5A). Furthermore, boyden chamber assays showed that BLU9931 did not alter the migration of PK-1 cells (Figure 5B). Thus, these results indicate that FGF19/FGFR4 signaling does not regulate PDAC cell migration. By contrast, invasion assays showed that numbers of invaded cells were significantly reduced in 2 μM BLU9931-treated PK-1 cells compared with control cells (Figure 5C), indicating that autocrine/paracrine FGF19/FGFR4 signaling promotes invasion. One of key features of invasion is increased production of matrix metalloproteinases (MMPs) [46,47] and MMP2, MMP9, and MT1-MMP are known to be expressed in PDAC cells [48]. Therefore, we hypothesized that FGF19/FGFR4 signaling might promote MMP expression and activity. Real-time qPCR revealed that MT1-MMP mRNA levels were significantly reduced in BLU9931-treated PK-1 cells, while MMP2 mRNA levels were upregulated (Figure 5D). Furthermore, Western blot analysis showed that protein levels of MT1-MMP were significantly reduced in BLU9931-treated PK-1 cells (Figure 5E). MT1-MMP is well known to induce activated MMP2 by proteolytic cleavage of prodomain of proMMP2 [46,47]. Therefore, we next examined the activity of MMP2 released from BLU9931-treated PK-1 cells using gelatin zymography. As shown in Figure 5F, collagenase activity of MMP2 was reduced in BLU9931-treated PK-1 cells. A BLU9931-induced reduction in invasion, MT1-MMP expression, and MMP2 activity was also observed in T3M-4 cells (Figure S4). Thus, autocrine/paracrine FGF19/FGFR4 signaling regulates expression of MT1-MMP and contributes to the activation of MMP2 in PDAC cells.

### 2.5. Induction of Senescence in PDAC Cells by Long-Term Treatment with BLU9931

**Figure 6:**
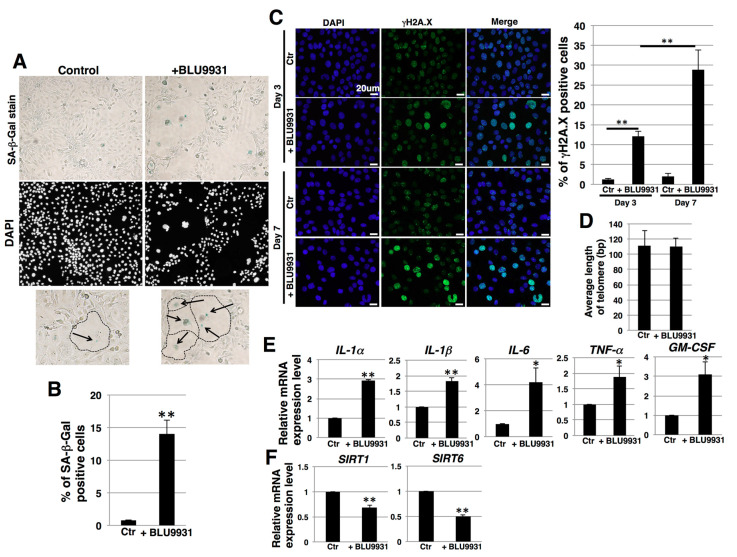
Induction of senescence in PDAC cells by long-term incubation with BLU9931. (**A**) PK-1 cells that were incubated with or without 2 μM BLU9931 for 7 days were stained for SA-β-Gal activity. Representative images of staining for SA-β-Gal and DAPI are shown. Regions surrounded by dotted lines in the lower panel show representative senescent cells that are highlighted by arrows. (**B**) SA-β-Gal-positive cells in (**A**) were quantitated as a percentage of total cell numbers. Representative results from measurements of 4 fields are shown. (**C**) Immunocytochemical staining performed in PK-1 cells that were incubated with or without 2 μM BLU9931 for 3 or 7 days. Representative images are shown (γH2A.X, green; DAPI, blue). The associated histogram shows the percentage of γH2A.X-positive cells (mean ± SD) from four fields. (**D**) Telomere length assay by real-time PCR in PK-1 cells incubated with or without 2 μM BLU9931 for 7 days. Results are the means ± SD from three independent experiments. (**E**) Real-time qPCR analysis of SASP factors in PK-1 cells that were incubated with or without 2 μM BLU9931 for 7 days. Results shown are normalized to values obtained for control cells (value = 1). Results are the means ± SD from three independent experiments. (**F**) Real-time qPCR analysis of *SIRT1* and *SIRT6* in PK-1 cells that were incubated with or without 2 μM BLU9931 for 7 days. Results shown are normalized to values obtained for control cells (value = 1). Results are the means ± SD from three independent experiments. * *p* < 0.05, ** *p* < 0.01. Control (Ctr): Control cells were incubated with dimethyl sulfoxide.

During long-term culture (one week) with BLU9931, some populations of cells exhibited an enlarged and flattened morphology (Figure 6A). Such changes may occur in senescent cells [49]. Therefore, we next examined the effects of BLU9931 on senescence in PK-1 cells by assaying for senescence-associated β-galactosidase (SA-β-Gal) activity, which is a well-known marker for senescent cells. BLU9931 increased the number of SA-β-Gal-positive cells compared with the control group (Figure 6A,B). By contrast, a 3-day incubation with BLU9931 did not alter SA-β-Gal staining in PK-1 cells (Figure S5A,B). Furthermore, phosphorylation of H2A.X (γH2A.X), which is an early sign of DNA damage as well as a senescence marker [50,51], was increased in PK-1 cells that had been incubated for 3 days with BLU9931 and further increased in long-term culture with BLU9931 (Figure 6C). There were no differences in telomere length between control and long-term BLU9931-treated PK-1 cells (Figure 6D). Taken together, these results suggest BLU9931 induces premature senescence through DNA damage, possibly through the ATM pathway.

Senescent cells may exhibit a senescence-associated secretory phenotype (SASP) [52], a process whereby high levels of SASP factors, including inflammatory cytokines, are secreted. In the present study, prolonged incubation of PK-1 cells with BLU9931 resulted in a significant increase in the levels of SASP-associated cytokines (IL-1α, IL-1β, IL-6, TNFα, and GM-CSF) when compared with the corresponding levels in control cells (Figure 6E). In T3M-4 cells, the number of SA-β-Gal-positive cells accompanying enlarged and flattened morphology was also increased following prolonged incubation with BLU9931, but the percentage of SA-β-Gal-positive cells was lower than in PK-1 cells (Figure S5C,D). Moreover, among the above SASP cytokines, only GM-CSF was significantly upregulated in these T3M-4 cells (Figure S5E). 

It is known that reduction of sirtuin1 (SIRT1) and SIRT6 leads to induction of premature senescence caused from attenuation of DNA damage repair [53]. We therefore examined the expression of SIRT1 and SIRT6 in relation to the induction of premature senescence by BLU9931. BLU9931 reduced the expression of SIRT1 and SIRT6 (Figure 6F and Figure S5F), suggesting that long-term BLU9931 treatment induces premature senescence in PDAC cells, in part, through downregulation of SIRT1 and SIRT6.

### 2.6. Effects of Senolytic Drugs on BLU9931-Induced Senescent PDAC Cells

**Figure 7:**
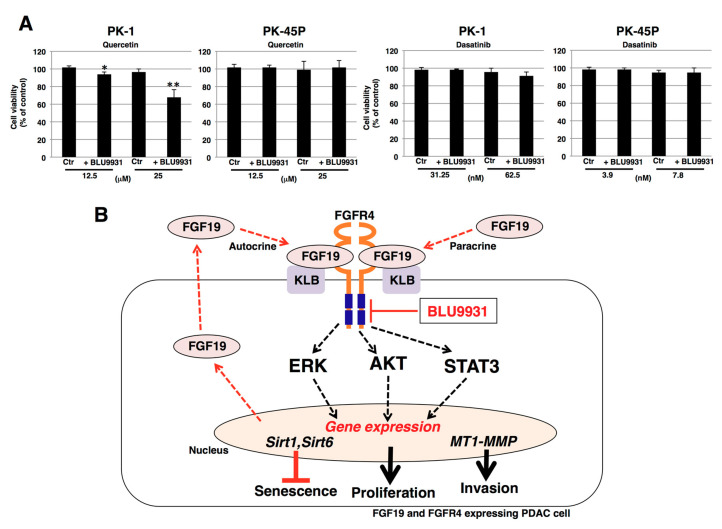
Effects of senolytic drugs on BLU9931-induced senescent PDAC cells. (**A**) All cells were incubated for 7 days in the presence or absence of 2 μM BLU9931. PK-1 cells were then incubated for 4 days with quercetin (12.5 or 25 μM) or dasatinib (31.25 or 62.5 nM) whereas PK-45P cells were incubated for 4 days with quercetin (12.5 or 25 μM) or dasatinib (3.9 or 7.8 nM). Cell viability was then measured by ATP assays. Results are the means ± SD from three independent experiments. * *p* < 0.05, ** *p* < 0.01. Control (Ctr): Control cells were incubated with DMSO. (**B**) Schematic representation of autocrine/paracrine FGF19/FGFR4 signaling in PDAC cells. Klotho-β (KLB) binding to FGFR4 enables FGF19 binding to the FGFR4-KLB heterodimer and promotes FGFR4 dimerization, thereby activating downstream signaling, such as ERK, AKT, and STAT3. These pathways promote cell proliferation, upregulate membrane-type matrix metalloproteinase-1 (MT1-MMP) that contributes to enhanced cell invasion, and upregulate sirtuin1 (SIRT1) and SIRT6 to help suppress senescence. Additionally, FGF19 auto-induces FGF19 expression, thereby promoting an autocrine/paracrine loop for persistent FGF19-mediated activation of FGFR4. BLU9931, a highly specific and irreversible FGFR4 inhibitor, inhibits the tyrosine kinase activity of FGFR4 by interacting with cysteine residue 552 near FGFR4’s ATP binding region, thereby suppressing downstream signaling and impeding upregulation of the above genes.

Quercetin and dasatinib are senolytic drugs [54] that can selectively eliminate senescent cells. Therefore, the dose-dependent effects of quercetin or dasatinib on the viability of PDAC cell lines (PK-1, PK-45P, T3M-4) were examined next. We added increasing concentrations of quercetin (1.56–50 μM) or dasatinib (7.8–500 nM) to PK-1, PK-45P, and T3M-4 cells. As shown in Figure S6, we identified the maximal concentration without cytotoxic effects as follows: quercetin (25 μM) and dasatinib (62.5 nM) for PK-1 cells, quercetin (25 μM) and dasatinib (7.8 nM) for PK-45P cells, and quercetin (12.5 μM) and dasatinib (7.8 nM) for T3M-4 cells. We then assessed the cytotoxic effects of senolytic drugs on BLU9931-induced senescent PDAC cells. Quercetin significantly decreased the viability of long-term BLU9931-treated PK-1 and T3M-4 cells (Figure 7A and Figure S6) but not PK-45P cells (Figure 7A), whereas dasatinib was not effective in any of the BLU9931-treated cells (Figure 7A and Figure S7). Thus, the senolytic drug, quercetin, was effective in those PDAC cells in which BLU9931 induced senescent changes. As shown in Figure S8A, the poly ADP ribose polymerase (PARP) inhibitor olaparib inhibited the growth of PK-1 cells dose dependently, with an IC50 of 8 μM. At this concentration, olaparib did not significantly alter the number of apoptotic and necrotic/injured cells that had been incubated with BLU9931 (Figure S8B). Next, we examined senescence and cytotoxic effects of BLU9931+quercetin+olaparib. BLU9931+olaparib enhanced the proportion of senescent cells compared with BLU9931 alone (Figure S9A,B). This was associated with a profound decrease in the total number of cells, indicating that in addition to inducing senescence, olaparib inhibited proliferation and exerted a senolytic effect in these cells. However, there was no additive effect on PK-1 cell viability with quercetin addition in BLU9931+olaparib-treated cells (Figure S9C). We speculate that senescent cells induced by olaparib treatment are not sensitive to quercetin.

## 3. Discussion

Downstream pathways of FGF19/FGFR4 signaling, such as PI3K-AKT and MEK-ERK, lead to enhanced cell proliferation and survival in cancer cells. The selective FGFR4 inhibitor BLU9931 that was used in this study can inhibit FGF19/FGFR4-positive hepatocellular carcinoma tumors [35]. BLU9931 also exerts potent anticancer effects in head and neck cancers, breast cancer, colorectal cancer, and squamous cell carcinomas [34,55,56,57]. Moreover, in breast cancer, autocrine FGF19/FGFR4 signaling is important for cell survival, and FGF19/FGFR4 co-expression was observed in primary breast tumors [56]. In the current study, we demonstrated that BLU9931 was also effective at inhibiting the proliferation of PDAC cells (Figure 3 and Figure S3). Furthermore, we found that *FGF19* expression was lowered following inhibition of FGF19/FGFR4 signaling (Figure 3D and Figure S3C), suggesting that in PDAC cells, the FGF19/FGFR4 axis controls FGF19 in a positive loop and that FGF19 has the potential to act in an autocrine/paracrine manner when produced and released by these cells. Indeed, in two of our tested PDAC cell lines, PK-1 and T3M-4, *FGF19* (Figure 1F) and FGFR4 (Figure 1C–E) expression was relatively elevated. Moreover, activation of FGFR4 is associated with *FGF19* gene amplification in several types of solid tumors [58,59].

In the present study, we also demonstrated that a subset of PDAC samples harbored an 11q13.3 amplicon and exhibited *FGF19* gene amplification in conjunction with amplification of other genes within this amplicon, including *FGF3, FGF4, CCND1, RAD9A, RPS6KB2, GAB2*, and *PAK1*. In three cases, there was also co-amplification of *FGF19* with *FGFR4* even though the *FGFR4* gene is located on chromosome 5q25. Altogether, 27 of the 109 samples in the UTSW-TCGA data set exhibited concomitant increases in FGF19 and FGFR4 expression. Moreover, in three of the cases, *FGFR4* co-amplified with *FGFR3*, a gene located on 4p16.3. Importantly, in a genomic study of 4853 solid cancers, 7.1% were found to have *FGFR* gene alterations, 66% of which consisted of gene amplifications [59], underscoring the importance of FGFR signaling in cancer pathobiology. Taken together, these observations suggest that the FGF19/FGFR4 may activate a novel driver oncogene pathway in PDAC. Additional lines of evidence in support of this possibility derive from our observation that 49% of PDAC samples analyzed in the present study exhibited strong FGFR4 immunoreactivity, that autocrine/paracrine FGF19/FGFR4 signaling was functional in PK-1 and T3M-4 cells that expressed relatively high levels of FGF19 and FGFR4, and that FGFR4 downstream signaling was readily inhibited by BLU9931, a highly specific inhibitor of FGFR4 that does not target other FGFRs or other tyrosine kinase receptors.

The FGF19/FGFR4 signaling is known to promote invasion and metastasis in several different types of cancer [57]. In the current study, we demonstrated for the first time that FGF19/FGFR4 signaling is involved in PDAC cell invasion through the regulation of MT1-MMP expression and MMP2 activity in these cells (Figure 5 and Figure S4). Our findings are thus consistent with the known strong correlation between MT1-MMP expression and cancer cell invasiveness [60]. Moreover, MMP2 activity and effects on cellular invasion are MT1-MMP dependent [61]. It is therefore possible that the BLU9931-mediated decrease in MT1-MMP levels led to the suppression of invasion, in part, as a result of the associated decrease in MMP2 activity. In as much as STAT3 directly controls expression of MT1-MMP [62], inhibition of the FGF19/FGFR4-activated STAT3 pathway by BLU9931, as evidenced by marked suppression of STAT3 phosphorylation, suggests that BLU9931 may also suppress metastasis in PDAC. Given the important role of the metastatic process in PDAC, it will be important to test this possibility in mouse models of PDAC metastasis.

PNETs overexpressing FGFR4 exhibit an increased propensity for forming hepatic metastases in mice [29] and hepatocellular carcinoma in humans is associated with excessive FGFR4-FGF19 signaling [31,35]. Our analysis of TCGA data revealed that 47 of 109 patients with PDAC overexpressed FGF19, and in 11 of these patients, this was due to *FGF19* gene amplification (Figure 2A). It is therefore possible that high levels of FGF19 in ~43% of patients with PDAC are released by their tumors and drain directly into the liver via the pancreatic vein, thereby facilitating the growth of metastases within the FGF19-enriched hepatic microenvironment. In this context, the potential of senolytic therapy with newer and safer agents, such as BLU554 [63], which is currently undergoing a large phase 1 clinical trial in hepatocellular carcinoma patients (NCT02508467), may provide an exciting therapeutic opportunity in patients with PDAC and could also help treat their hepatic metastases.

To date, the possible involvement of FGF19/FGFR4 signaling in cancer stem cells (CSCs) has not been definitively established. Our findings suggest that FGF19/FGFR4 signaling had little association with stemness in PDAC cells (Figure 4). Thus, *FGFR4* and *FGF19* expression was reduced in 3-D culture (Figure 4E), which is an optimal culture condition for CSCs [43,44,45], suggesting that FGF19/FGFR4 activation was not contributing to CSC formation. FGFR1 and FGFR4 are known to compete via their interactions with KLB [58]. Moreover, in FGFR1-amplified non-small cell lung cancer cells, FGFR1 signaling contributes to maintenance of the CSC phenotype [64]. Furthermore, PD173074, a selective FGFR1 inhibitor, suppressed the CSC-like phenotype of PDAC cells [65]. Therefore, it is possible that another pathway, such as FGF21/FGFR1, may be involved in modulation of stemness through KLB in PDAC cells. Moreover, CSCs can undergo epithelial-mesenchymal transition (EMT), and FGF19/FGFR4 signaling has been implicated in this process in colorectal cancer and hepatocellular carcinoma [66,67]. However, the actions of endocrine FGFs are complex and may be context dependent [68]. For example, the pancreatic acinar cell expresses high levels of FGF21 that are suppressed by oncogenic Kras, and placing such mice on a high-fat diet promotes pancreatic inflammation and PanIN progression to PDAC, processes that are attenuated by the exogenous administration of FGF21 [69]. Moreover, acinar cells mostly express the 3-Ig form of FGFR1, whereas the PDAC cells express the 2-Ig form of FGFR1 [70], which leads to enhanced constitutive FGFR1 signaling [71]. Given this complexity, further studies are necessary to improve our understanding of the relationship between FGF19/FGFR4 signaling, CSCs, and EMT in PDAC.

Cellular senescence is a complex biological process, whereby cell proliferation is arrested in response to various stressors [52]. Senescence can also occur in cancer cells, which can exhibit context-dependent biological alterations, such as dormancy and suppression of tumor progression or exit from dormancy with CSC-like features and enhanced capacity to invade and metastasize [72,73,74,75]. Senescent tumor cells are also observed following chemotherapy or radiation therapy [76]. While the induction of cancer cell senescence, per se, has been proposed as a therapeutic strategy in tumor therapy, the persistent presence of senescent cancer cells within the tumor microenvironment (TME) can promote cancer recurrence due to TME remodeling that includes recruitment of myeloid-derived suppressor cells (MDSCs) and chronic SASP that promote senescence bypass mechanisms [77]. Thus, combining senescence-inducing therapy with senolytic drugs could be beneficial to short- and long-term outcomes in cancer patients [78,79]. Here, we showed that BLU9931, a highly selective inhibitor of FGF19/FGFR4 signaling, induces senescence in some cases, and further that the senolytic drug, quercetin, is effective on senescent PDAC cells (Figure 6, Figure 7, Figures S5 and S7). Quercetin exerts multiple effects in different cancers, such as targeting BCL-2 family members, hypoxia inducible factor-1α, and PI3-kinase, and p21-related anti-apoptotic pathways [80]. By contrast, dasatinib exerts more limited effects, such as targeting Src family kinases and other key senescent cell anti-apoptotic pathway elements [44]. In addition to regulating different targets, quercetin and dasatinib may exert their effects more efficiently in different cell types. For example, quercetin targets senescent endothelial cells but not senescent adipocyte progenitors, while dasatinib targets senescent adipocyte progenitors but not senescent endothelial cells [54]. Thus, the effect of quercetin alone on PDAC cells may be due to the difference in the mechanisms of action between quercetin and dasatinib.

The sirtuins are members of a family of deacetylases (SIRT1 through SIRT7) that modulate numerous cellular processes [53]. SIRT1 and SIRT6 are nucleus-localized sirtuins that also modulate cellular senescence, and downregulation of SIRT1 and SIRT6 has been demonstrated to induce premature senescence in a variety of cell types [81,82,83]. In the present study, we determined that BLU9931 reduced *SIRT1* and *SIRT6* levels, leading to the induction of premature senescence in PDAC cells (Figure 6). It is not clear, however, whether inhibition of SIRT1 or SIRT6, per se, was sufficient to cause the observed effects on senescence since rescue experiments to restore SIRT1 or SIRT6 activity and/or expression were not carried out in the present study. Nonetheless, quercetin but not dasatinib is known to inhibit SIRT6 activity [84]. It is possible, therefore, that due to its actions on SIRT6, quercetin was able to enhance the effects of BLU9931.

Our analysis of the effects of BLU9931 on cell cycle-regulating genes revealed that in responsive PDAC cells, BLU9931 upregulated *p27* and *p21*, both of which slow down progression through the G1 phase of the cell cycle, and downregulated *CDK1*, which contributes to the cell’s ability to progress through the G1/S and G2/M phases of the cell cycle. The net effect is that BLU9931 attenuates cell cycle progression as shown in Figure 3C. Moreover, BLU9931 upregulated *E2F1* expression. Elevated E2F1 levels can promote cell cycle arrest and/or apoptosis [85], and the possibility that BLU9931 may activate this pathway warrants further studies.

Our current findings suggest that BLU9931 may have a therapeutic potential in patients with PDAC and that a precision medicine-based strategy could be devised based on FGF19/FGFR4 expression and downstream signaling analysis. In addition, therapy with BLU9931 could lead to novel treatment strategies in PDAC perhaps via the sequential addition of senolytic drugs, such as quercetin, its analogs, or the PARP inhibitor olaparib, all of which could have fewer side effects than anticancer drugs. Importantly, BLU9931 also upregulated the expression of GM-CSF, which can promote pancreatic cancer progression and metastasis [86,87]. However, FGFR4 can also activate pro-metastatic signaling [88] and can crosstalk with EGFR by promoting amphiregulin expression [89]. Moreover, EGFR activation is essential for oncogenic Kras-driven PanIN progression to PDAC [90]. It will be important, therefore, to determine whether the combination of BLU9931 and senolytic agents will abrogate the upregulation of GM-CSF expression and suppress the metastatic potential of PDAC cells, and to assess the need for inhibiting GM-CSF actions in any therapeutic trials for PDAC with BLU9931.

## 4. Materials and Methods 

### 4.1. Immunohistochemistry

Paraffin-embedded sections from pancreas tissue microarrays (PA1002, PA2072; US Biomax, Inc. Rockville, MD, USA) were immunostained using Histofine Simple Stain MAX PO (R) kit. After deparaffinization, antigen retrieval was performed at 121 °C for 15 min in a sodium citrate buffer solution (pH 6.0). Then, endogenous peroxidase activity was blocked by incubating sections for 30 min with 0.3% hydrogen peroxide in methanol. The tissue sections were incubated overnight with the rabbit polyclonal anti-FGFR4 antibody (1:100 in dilution; Sigma-Aldrich, St. Louis, MO, USA). Bound antibodies were detected with Simple Stain MAX PO (R) reagent, using diaminobenzidine-tetrahydrochloride chromogen (DAB) as the substrate. Negative control studies were performed by omitting the primary antibodies. For the evaluation of the intensity of FGFR4 immunostaining in the tissue microarray, the following scale was employed: 0, no staining; 1+, mild staining; 2+, moderate staining; and 3+, intense staining. Two pathologists (H. Y. and Y. M.) independently evaluated the staining results. Scales 0 and 1 were low, and 2 and 3 were divided into FGFR4 highly localized groups. Correlation between FGFR4 expression and tumor grades were analyzed in 129 cases, and the other factors were analyzed in 136 cases, because of the provided clinical data from the tissue arrays. Microscopic images were taken using Mantra, multi-spectral microscopy (PerkinElmer, Waltham, MA, USA), and then images were loaded into inform software ver. 2.4 (PerkinElmer) to separate specific chromogens, DAB, and hematoxylin, by using the spectral compensation library built in the software according to the manufacturer’s instruction.

### 4.2. Cell Culture

The human PDAC cell lines PK-1, PANC-1, PK-59, and MIA PaCa-2 were obtained from the Cell Resource Center for Biomedical Research, Institute of Development, Aging and Cancer, Tohoku University (Sendai, Japan). PK-45P, PK-8, T3M-4, and KP4 human PDAC cells were provided by the RIKEN BRC through the National Bio-Resource Project of the MEXT, Japan. Cells were grown in growth medium (RPMI 1640 medium containing 10% fetal bovine serum) at 37 °C under a humidified 5% CO_2_ atmosphere. For 3-D culture, cells in growth medium were plated at 1.0 × 10^4^ cells/well or 3.0 × 10^3^ cells/well in 24-well ultra-low attachment plates (Corning Inc., Kennebunk, ME, USA) or 96-well ultra-low attachment plates (Thermo Fisher Scientific, Waltham, MA, USA), respectively. The spheres were aspirated after 7 days using micropipettes and placed in microcentrifuge tubes for use in further experiments. Cells were treated with BLU9931 (EMD Millipore Corp., Burlington, MA, USA) or 0.005% dimethyl sulfoxide as vehicle control for 3 days, and after further experiments were performed.

### 4.3. Genotyping

Genomic DNA was extracted from PDAC cell lines using DNeasy Blood & Tissue Kit (QIAGEN, Hilden, Germany) according to the manufacturer’s instructions. Genotypes were determined according to the previous report [91]. Briefly, *FGFR4* exon 9 was amplified using primers (Forward: 5′-GAC CGC AGC AGC GCC CGA GGC CAG-3′; Reverse: 5′-AGA GGG AAG AGG GAG AGC TTC TG-3′) and analyzed for Gly388Arg polymorphism. PCR products were digested with *BstNI* (New England Biolabs Inc., Ipswich, MA, USA). SNP Arg388 in *FGFR4* gene was characterized by two distinctive fragments of 82 and 27 bp. On the other hand, the *FGFR4* Gly388 wild-type allele was identified by a single fragment of 109bp.

### 4.4. FACS Analysis 

Cells were harvested and dissociated cells were incubated on ice for 30 min with rabbit polyclonal anti-FGFR4 antibody (antibodies-online GmbH, Aachen, Germany) diluted in FACS buffer (0.5% [*w*/*v*] bovine serum albumin and 0.1% [*w*/*v*] sodium azide in phosphate buffered saline [PBS]). After washing, the cell suspension was incubated on ice for 30 min with Alexa Fluor^®^ 488-conjugated secondary antibodies (Molecular Probes, Eugene, OR, USA) diluted in FACS buffer. Cell analysis was performed using a FACSAria™ Cell Sorter (Becton Dickinson, Franklin Lakes, NJ, USA). Mean fluorescence intensities (MFIs) were calculated after subtracting the intensities of the controls.

### 4.5. Real-Time qPCR

Total RNA was isolated from cells using the RNeasy plus mini kit (QIAGEN) and subsequently reverse-transcribed using the ReverTra Ace^®^ qPCR RT Kit (Toyobo, Osaka, Japan) according to the manufacturer’s instructions. Real-time qPCR was performed using the Power Sybr^®^ Green kit (Applied Biosystems, Foster City, CA, USA) and a StepOnePlus^™^ real-time PCR system (Applied Biosystems). *β-actin* was amplified as an internal control. Primer sets for real-time qPCR are listed in Table S1. Real-time qPCR analysis for *FGFR4* was performed using TaqMan Fast Universal PCR Master Mix (Life Technologies Corporation, Carlsbad, CA, USA) and TaqMan Gene Expression Assays (Life Technologies), with 18S rRNA serving as an internal control.

### 4.6. Cell Proliferation Assay

Cells were cultured in growth medium at a density of 3 × 10^3^ cells/well in 96-well plates followed by incubation for 72 h. ATP assays were used to examine proliferation using the CellTiter-Glo^®^ 2.0 Assay (Promega, Madison, WI, USA) according to the manufacturer’s protocol.

### 4.7. Apoptosis Assay

Measurement of the percent of apoptotic cells was performed using an Annexin V-Biotin Apoptosis Detection kit (BioVision Inc., Milpitas, CA, USA) per the manufacturer’s instructions. Cells were analyzed using a FACSAria™ Cell Sorter.

### 4.8. Cell Cycle Assay

Cells were washed in PBS, resuspended in PBS, and stained with cell cycle assay solution (deep red; Dojindo Molecular Technologies, Inc., Rockville, MD, USA) at 37 °C for 15 min. The cell cycle profiles were obtained using a FACSAria™ Cell Sorter at 640 nm. Data were analyzed using FlowJo software (Becton Dickinson).

### 4.9. Immunoblotting

Cells were lysed with lysis buffer (50 mM Tris-HCl pH 7.4, 150 mM NaCl, 1.5 mM MgCl_2_, 5 mM EDTA, and 1% Triton^™^ X-100) containing protease and phosphatase inhibitor cocktails. Samples prepared as described above were separated by SDS-PAGE and then transferred onto Polyvinylidene difluoride membranes (Merck Millipore, Billerica, MA, USA). After blocking, the membranes were incubated with the following primary antibodies: polyclonal rabbit anti-FGFR4 (ab41948; Abcam, Cambridge, UK), monoclonal rabbit anti-ERK1/2 (#4695; Cell Signaling Technology, Danvers, MA, USA), monoclonal rabbit anti-pERK1/2 (#4370; Cell Signaling Technology), monoclonal rabbit anti-AKT (#4691; Cell Signaling Technology), monoclonal rabbit anti-pAKT (#4060; Cell Signaling Technology), monoclonal mouse anti-STAT3 (#9139; Cell Signaling Technology), polyclonal rabbit anti-pSTAT3 (#9131; Cell Signaling Technology), monoclonal rabbit anti-MT1-MMP (ab51074; Abcam), and monoclonal mouse anti-β-actin (A5316; Sigma-Aldrich). Membranes were then incubated with the appropriate peroxidase-conjugated secondary antibodies (Cell Signaling Technology), washed, and developed with ECL^™^ Prime reagents (GE Healthcare, Piscataway, NJ, USA). Image densitometry was performed with ImageJ software (National Institutes of Health, Bethesda, MD, USA). Full blot images with molecular weight markers are shown in Figure S10.

### 4.10. Sphere-Forming Assay 

To form spheres, cells (5.0 × 10^3^ cells/well) were plated in 24-well ultra-low attachment plates with RPMI 1640 medium containing FGF-2 (10 ng/mL, ReproCELL, Tokyo, Japan) and EGF (20 ng/mL, AUSTRAL Biologicals, San Ramon, CA, USA). After 7 days, the spheres that formed were counted by using a phase-contrast microscope (Eclipse TS-100, Nikon, Tokyo, Japan).

### 4.11. Anti-Drug Resistance Assay

Cells (3.0 × 10^3^ cells/well) were plated in 96-well culture plates with growth medium with or without BLU9931. Each anticancer drug was administered at the indicated concentration after 3 days culture and cell growth rates were measured by ATP assays 4 days after treatment of anticancer drug. Cell viability was calculated as the percentage of luminescence in drug-treated cells relative to non-treated control cells.

### 4.12. Wound Healing/Cell Scratch Assay

Cells were seeded at a density of 7 × 10^3^ cells on each side of an Ibidi culture insert (Ibidi GmbH, Martinsried, Germany), with a 500-μm separation between each side of the well, and allowed to grow for 24 h in growth medium. After removal of the insert, cells were incubated in the same medium. Cells were photographed using phase contrast microscopy at insert removal (0 h) and following 6 and 24 h of incubation. Accurate measurements of the wounds were taken during the time course to calculate the migration rate according to the equation: Percentage of wound healing = [(wound area at 0 h) − (wound area at 6 or 24 h)]/(wound length at 0 h) × 100.

### 4.13. Boyden Chamber Assay

Cell culture inserts (8-μm pore size and 6 mm in diameter) were used according to the manufacturer’s instructions. Cells were plated at a density of 1 × 10^5^ cells/500 μL on the upper chamber of the inserts with serum-free medium, and medium containing 10% serum was used in the lower chamber. Six hour later, cells that had migrated through the membrane pores were fixed and stained with a Diff-Quick staining kit (Polysciences, Inc., Warrington, PA, USA), and counted under a light microscopy.

### 4.14. Invasion Assay

Invasion assays were performed using Corning Matrigel invasion chambers (pore size: 8 μm, Discovery Labware Inc., Bedford, MA, USA). Cells were plated at a density of 1 × 10^5^ cells/500 μL on the upper surface of the inserts, and, 16 or 24 h later, cells that had migrated through the membrane to the lower surface of the filter were fixed and stained with a Diff-Quick staining kit (Polysciences, Inc.) and counted under a light microscope.

### 4.15. MMP Gelatin Zymography

Media samples collected from cells cultured in serum-free medium for 24 h at 37 °C were concentrated using Amicon^®^ Ultra Centrifugal Filters (Merck Millipore), and protein assays were performed. The same amounts of samples were resolved on 8% SDS-PAGE gels containing 4.0 mg/mL gelatin. The gels were rinsed with wash buffer (50 mM Tris-HCl, pH 7.5, 5 mM CaCl_2_, 1 μM ZnCl_2_, 2.5% Triton X-100) and soaked in incubation buffer (50 mM Tris-HCl, pH 7.5, 5 mM CaCl_2_, 1 μM ZnCl_2_, 1% Triton X-100) at 37 °C for 24 h. After incubation, the gels were fixed and stained with Coomassie R-250, washed, and scanned.

### 4.16. SA-β-Gal Assay

SA-β-Gal activity was assayed using a senescence detection kit (BioVision Inc., Milpitas, CA, USA) as described in our previous reports [92]. Briefly, cells were washed twice with PBS, exposed to fixation solution for 10 min, and then incubated overnight in freshly prepared staining solution. After staining, cells were counterstained with DAPI and then four fields of view were examined microscopically. By counting the number of SA-β-Gal-positive cells based on their blue color and the total number of cells stained with DAPI using ImageJ software, the percentage of SA-β-Gal-positive cells was calculated to estimate the percentage of senescent cells.

### 4.17. Telomere Length Assay

Telomere length was quantified by real-time PCR using a Telomere Length Quantification qPCR Assay Kit (ScienCell Laboratory, Carlsbad, CA, USA) according to the manufacturer’s protocol. Genomic DNA samples were extracted as described in Section 4.3.

### 4.18. Immunostaining

Cells were fixed with 4% (*w*/*v*) paraformaldehyde and washed. Subsequently, cells were permeabilized with 0.1% [*v*/*v*] Triton^™^ X-100 and blocked with PBS containing 1% (*w*/*v*) BSA and 5% (*v*/*v*) normal goat serum. After washing, cells were incubated with monoclonal mouse anti-γH2A.X (sc-517348; Santa Cruz Biotechnology, Dallas, TX, USA) at 4 °C overnight. After washing, cells were stained with Alexa Fluor^®^ 488-conjugated secondary antibodies (Molecular Probes), and then counterstained with DAPI. Immunofluorescence images were acquired using a confocal laser scanning microscope (Leica Microsystems, Wetzlar, Germany). For the quantification of γH2A.X-positive cells, the numbers of γH2A.X-positive cells with green color and the total numbers of cells stained with DAPI from four fields were counted and then the percentage of γH2A.X-positive cells was calculated.

### 4.19. Senolytic Drug Sensitivity Assay

Cells (3.0 × 10^3^ cells/well) that were incubated in the presence or absence of BLU9931 for 7 days were plated in 96-well culture plates containing the respective media. Thus, only cells previously incubated with BLU9931 contained the FGFR4 inhibitor. Each senolytic drug, Quercetin (Cayman Chemical, Ann Arbor, MI, USA) or Dasanitib (Cayman Chemical), was then added as indicated and cell growth rates were determined by the ATP assay 4 days later. Cell viability was calculated as the percentage of luminescence in drug-treated cells relative to non-treated control cells.

### 4.20. Statistical Analysis

Results were shown as means ± SD, and differences between two groups were compared using Student’s t test or Welch’s t test. One-way ANOVA was performed when comparing multiple groups. Chi-square and Fisher’s exact tests were used to analyze correlations between FGFR4 expression and clinicopathological features. 

Statistical analysis was performed with EZR (Saitama Medical Centre, Jichi Medical University) [93].

## 5. Conclusions

In this study, we demonstrated that inhibition of signal transduction pathways through ERK, AKT, and STAT3 pathways by the FGFR4 inhibitor BLU9931 inhibited PDAC cell proliferation and invasion, in part by downregulating MT1-MMP in autocrine/paracrine FGF19/FGFR4 signaling-positive PDAC cells (Figure 7B). Furthermore, downregulation of SIRT1 and SIRT6 by BLU9931 may have contributed to senolysis in those cells (Figure 7B). Thus, we propose that BLU9931 is a promising drug for the therapy of FGFR4-positive PDAC.

## Figures and Tables

**Table 1:** Clinicopathological parameters of FGFR4 in PDAC tissues.

Variables		FGFR4 Expression		
		Low (<2)	High (2~3)	*p*-Value
**Tumor Grade**				*0.100*
	Grade 1	15	8	
	Grade 2	33	42	
	Grade 3	19	12	
**Primary Tumor**				*<0.001*
	T1	2	0	
	T2	27	8	
	T3	39	53	
	T4	1	6	
**Regional Lymph Node**				*0.083*
	N0	62	53	
	N1	7	14	
**Stage**				*<0.001*
	Stage I	27	2	
	Stage II	34	45	
	Stage III	7	16	
	Stage IV	1	4	

**Table 2:** Genotyping of FGFR4 in PDAC cell lines.

PDAC Cell Lines	FGFR4 Genotype	FGFR4 Amino Acid (388)
MIA PaCa-2	G/G	Gly/Gly
PK-8	G/G	Gly/Gly
PK-1	G/A	Gly/Arg
PANC-1	G/A	Gly/Arg
PK-45P	G/A	Gly/Arg
PK-59	A/A	Arg/Arg
T3M-4	A/A	Arg/Arg
KP4	A/A	Arg/Arg

## Supplementary Materials

The following are available online at https://www.mdpi.com/2072-6694/12/10/2976/s1, Table S1: Primer list, Figure S1: Characteristic FGFR4 localization on tissue microarray, Figure S2: Analysis of SNP Gly388Arg, Figure S3: Effects of BLU9931 on T3M-4 cells, Figure S4: Effects of BLU9931 on T3M-4 cell invasion, Figure S5: Induction of senescence in T3M-4 cells by long-term treatment of BLU9931, Figure S6: Dose-dependent effects of quercetin or dasatinib on the viability of PDAC cell lines, Figure S7: Effects of senolytic drug on BLU9931-induced senescent T3M-4 cells, Figure S8: Effects of Olaparib on the growth and viability of PK-1 cells, Figure S9: Effects of quercetin on BLU9931 ± Olaparib-induced senescent PK-1 cells, Figure S10: Western blots.
