# Supplementary material for: FGFR4 Inhibitor BLU9931 Attenuates Pancreatic Cancer Cell Proliferation and Invasion While Inducing Senescence: Evidence for Senolytic Therapy Potential in Pancreatic Cancer
Source: Cancers (Basel). 2020 Oct 14;12(10):2976. doi: 10.3390/cancers12102976 (PMC7602396; doi:10.3390/cancers12102976)
Supplement: Supplementary file 1 [file cancers-12-02976-s001.pdf]

Supplemental Materials: FGFR4 inhibitor BLU9931 attenuates pancreatic cancer cell proliferation and invasion while inducing senescence – evidence for senolytic therapy potential in pancreatic cancer

Norihiko Sasaki, Fujiya Gomi, Hisashi Yoshimura, Masami Yamamoto, Yoko Matsuda, Masaki Michishita, Hitoshi Hatakeyama, Yoichi Kawano, Masashi Toyoda, Murray Korc and Toshiyuki Ishiwata

Table S1. Primers list.

Gene	Forward Primer	Reverse Primer
FGF19	ACTGTGCTTTCGAGGAGGAGAT	GTGCTTCTCGGATCGGTACAC
Oct3/4	GGAGGAAGCTGACAACAATGAAA	GGCCTGCACGAGGGTTT
Nanog	CCAAAGGCAAACAACCCACTT	CGGGACCTTGTCTTCCTTTTT
Sox2	TGCGAGCGCTGCACAT	TCATGAGCGTCTTGGTTTTCC
ALDH1	GAGCCCTTGCATTGTGTTAGC	CCATGGTGTGCAAATTCAACAG
Nestin	TCCTGCTGTAGATGCAGAGATCAG	ACCCTGTGTCTGGAGCAGAGA
CD24	TCCAACTAATGCCACCACCAA	GACCACGAAGAGACTGGCTGTT
CD44v9	AGCAGAGTAATTCTCAGAGCTT	TGCTTGATGTCAGAGTAGAAGT
⤚-actin	GGTCATCACCATTGGCAATGAG	TACAGGTCTTTGCGGATGTCC
ABCG2	TGGCTGTCATGGCTTCAGTACT	CATTATGCTGCAAAGCCGTAAA
ABCB1	TGACAGCTACAGCACGGAAG	TCTTCACCTCCAGGCTCAGT
ABCC1	GAGAGTTCCAAGGTGGATGC	AGGGCCCAAAGGTCTTGTAT
ABCC2	TACCAATCCAAGCCTCTACC	AGAATAGGGACAGGAACCAG
MT1-MMP	GAAGGATGGCAAATTCGTCTTC	AGGGACGCCTCATCAAACAC
MMP2	GCGGCGGTCACAGCTACTT	TTCAGACTTTGGTTCTCCAGCTT
MMP9	GGACGATGCCTGCAACGT	GTACTTCCCATCCTTGAACAAATACA
IL-1⤚	TGGAGGCCATCGCCAAT	AGGAAGCTAAAAGGTGCTGACCTA
IL-1⤚	GTCTGGTCCATATGAACTGAAAGCT	GGACATGGAGAACACCACTTGTT
IL-6	AAAAAGGCAAAGAATCTAGATGCAA	GTCAGCAGGCTGGCATTTGT
TNF-⤚	CCCAGGCAGTCAGATCATCTTC	GCTTGAGGGTTTGCTACAACATG
GM-CSF	GAGCATGTGAATGCCATCCA	TTCATTCATCTCAGCAGCAGTGT
SIRT1	TGCGGGAATCCAAAGGATAA	CAGGCAAGATGCTGTTGCA
SIRT6	TTTGTGGAAGAATGTGCCAAGT	ATGGTGCCCACGACTGTGT
FGF3	TTTGGAGATAACGGCAGTGGA	CGTATTATAGCCCAGCTCGTGGA
FGF4	GAGCAGCAAGGGCAAGCTCTA	ACCTTCATGGTGGGCGACA
CCND1	GCGAGGAACAGAAGTGC	GAGTTGTCGGTGTAGATGC
RAD9A	TCTGCCTATGCCTGCTTTCTCT	AGCGGAAGACAGACAGGAAAGAC
RPS6KB2	CTTCCAGACTGGTGGCAAACTCTA	CAGCGTGATCTCAGCCAGGTA

GAB2	CGAAGAGAACTATGTCCCTATGC	AGGGGCAGGACTGTTCGT
PAK1	CGTGGCTACATCTCCCATTT	AGGCTTCTTCTTCTGCTTCTC
KLB	GCAGTCAGACCCAAGAAAATACAGA	CCCAGGAATATCAGTGGTTTCTTC
p53	TCTCCCCAGCCAAAGAAGAA	CCACGGATCTGAAGGGTGAA
p21	TGGAGACTCTCAGGGTCGAAA	GCGTTTGGAGTGGTAGAAATCTG
p27	AGACTGATCCGTCGGACAGC	CACAGAACCGGCATTTGGG
CCND2	GGACATCCAACCCTACATGC	CGCACTTCTGTTCCTCACAG
CCNE1	AAATGGCCAAAATCGACAGG	CGAGGCTTGCACGTTGAGTT
CDK1	ACAGGTCAAGTGGTAGCCATGA	ACCTGGAATCCTGCATAAGCA
CDK2	TTCTCATCGGGTCCTCCACC	TCGGTACCACAGGGTCACCA
CDK4	CTGTGCCACATCCCGAACTG	GCCTCTTAGAAACTGGCGCA
CDK6	CCGAAGTCTTGCTCCAGTCC	GGGAGTCCAATCACGTCCAA
E2F1	ATGTTTTCCTGTGCCCTGAG	ATCTGTGGTGAGGGATGAGG
PCNA	CCTGCTGGGATATTAGCTCCA	CAGCGGTAGGTGTCGAAGC

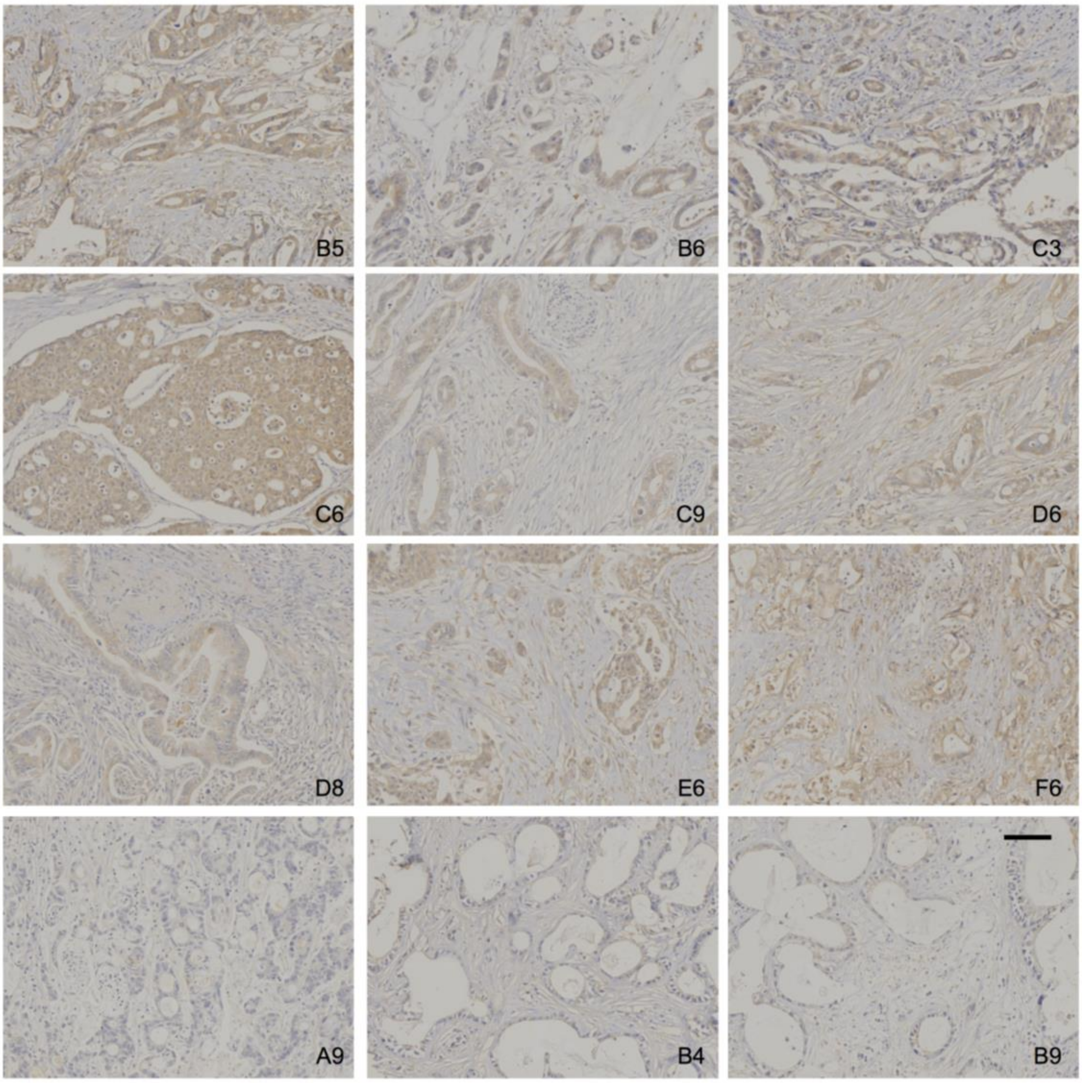

Figure S1. Characteristic localization of FGFR4 in human pancreatic cancer tissue microarray. Upper 9 panels exhibit strong FGFR4 immunoreactivity in the cancer cells, whereas the lowest 3 panels (A9,

B4, B9) show weak to absent FGFR4 immunoreactivity. Numbers shown in each panel indicate the core number of tissue microarray (PA1002). Scale bar: 100μm.

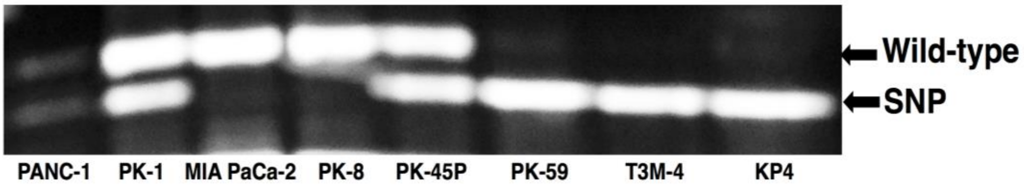

Figure S2. Analysis of SNP Gly388Arg. After digestion of PCR products with *BstN*, electrophoresis of each fragment was performed.

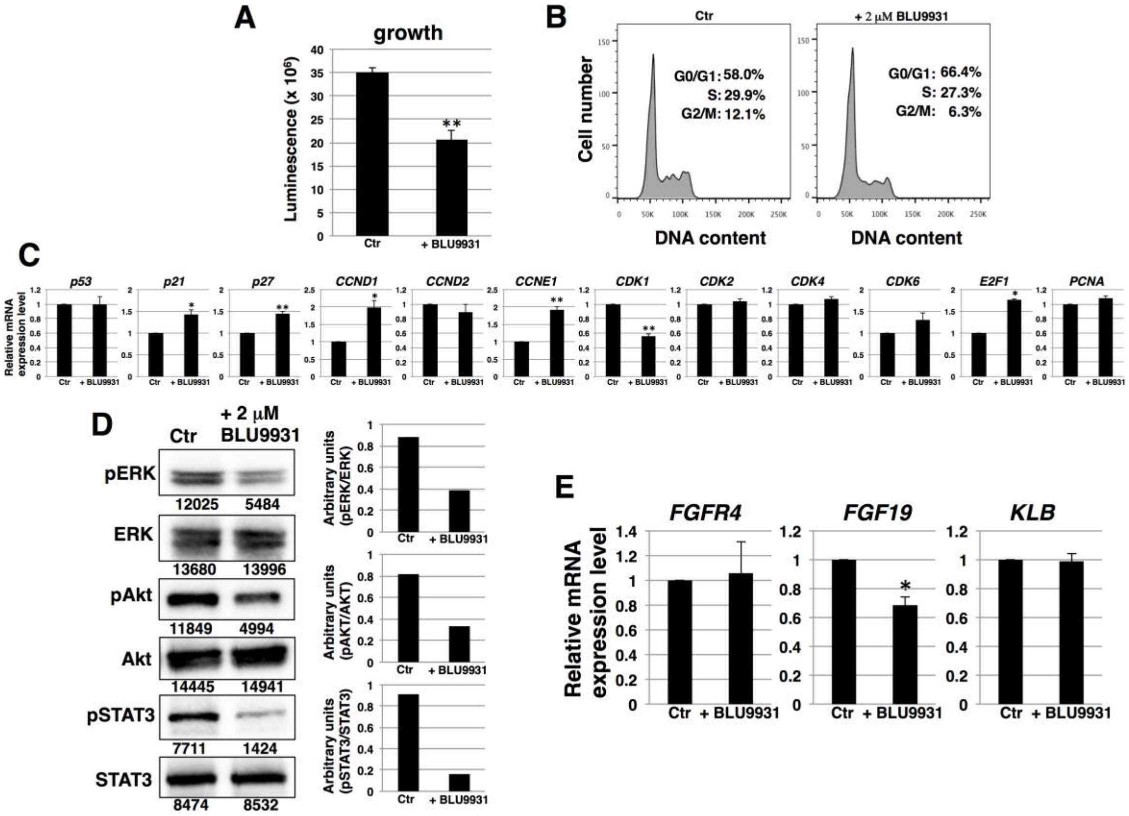

Figure S3. Effects of BLU9931 on T3M-4 cells. (**A**) T3M-4 cells were incubated with or without 2μM BLU9931 for three days and growth rates were determined. (**B**) Cell cycle analysis in T3M-4 cells that were incubated with or without 2μM BLU9931 for 3 days. (**C**) Real-time qPCR analysis of cell cycle related-genes in T3M-4 cells that were incubated with or without 2μM BLU9931 for 3 days. Results shown are normalized to values obtained for control cells (value = 1). (**D**) Western blot analysis for FGF19/FGFR4 signaling was performed in T3M-4 cells that were incubated with or without 2μM BLU9931 for 3 days. The expression of each band is shown under the blot. The histograms show mean densitometric readings for the phosphorylated proteins normalized to those of the loading controls. (**E**) Real-time qPCR analysis of *FGFR4, FGF19* or *KLB* in T3M-4 cells that were incubated with or without 2μM BLU9931 for 3 days. Results shown are normalized to values obtained for control cells (value = 1). *$p < 0.05$, **$p < 0.01$. Control (Ctr): Control cells were incubated with DMSO.

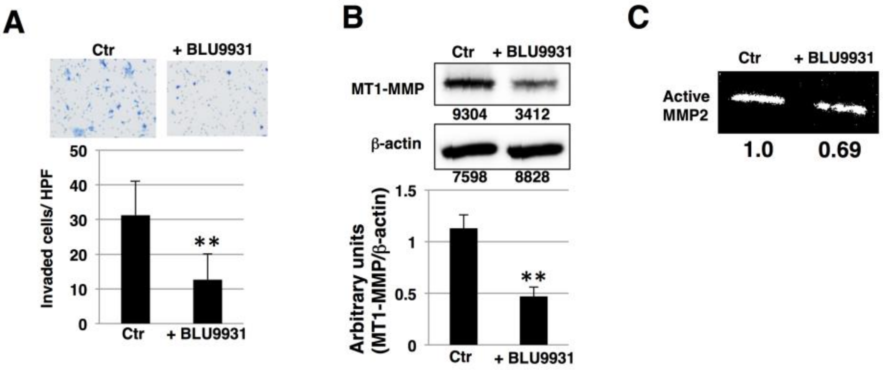

Figure S4. Effects of BLU9931 on T3M-4 cell invasion and senescence. (**A**) Matrigel invasion assays were performed in T3M-4 cells that were incubated with or without 2μM BLU9931 for 3 days. (**B**) Western blot analysis of MT1-MMP was performed in T3M-4 cells that were incubated with or without 2μM BLU9931 for 3 days. The expression of each band is shown under the blot. Histograms show mean densitometric readings ± SD for MT1-MMP normalized to those of the loading controls. (**C**) Gelatin zymography was performed using culture supernatants from T3M-4 cells that were incubated with or without 2μM BLU9931 for 3 days. Relative band intensity is shown. $^{**}p < 0.01$. Control (Ctr): Control cells were incubated with DMSO.

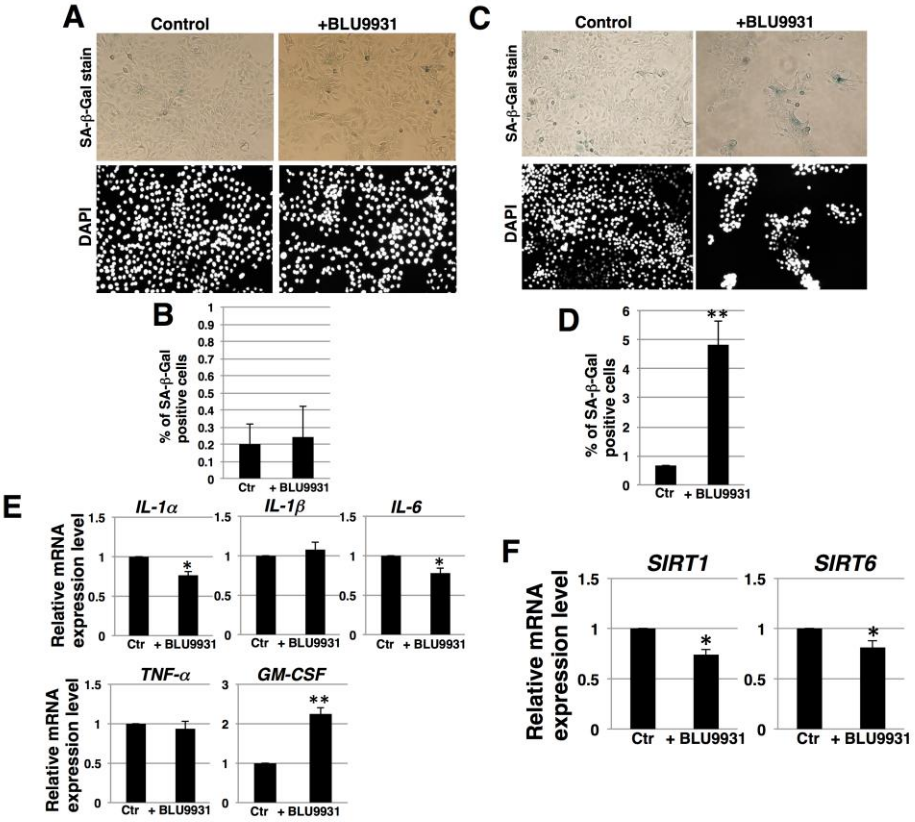

Figure S5. Induction of senescence in T3M-4 cells by long-term incubation with BLU9931. (**A**) PK-1 cells that were incubated with or without 2μM BLU9931 for 3 days were stained for SA-β-Gal activity. Representative images of SA-β-Gal and DAPI staining are shown. (**B**) SA-β-Gal-positive cells in (**A**) were quantitated as a percentage of total cell numbers. (**C**) T3M-4 cells that were incubated with or

without 2μM BLU9931 for 7 days were stained for SA-β-Gal. Representative images SA-β-Gal and DAPI staining are shown. (**D**) SA-β-Gal-positive cells in (**C**) were quantitated as a percentage of total cell numbers. (**E**) Real-time qPCR analysis of SASP-associated cytokines in T3M-4 cells that were incubated with or without 2μM BLU9931 for 7 days. Results shown are normalized to values obtained for control cells (value = 1). (**F**) Real-time qPCR analysis of *SIRT1* and *SIRT6* in T3M-4 cells that were incubated with or without 2μM BLU9931 treatment for 7 days. Results shown are normalized to values obtained for control cells (value = 1). $*p < 0.05$, $**p < 0.01$. Control (Ctr): Control cells were incubated with DMSO.

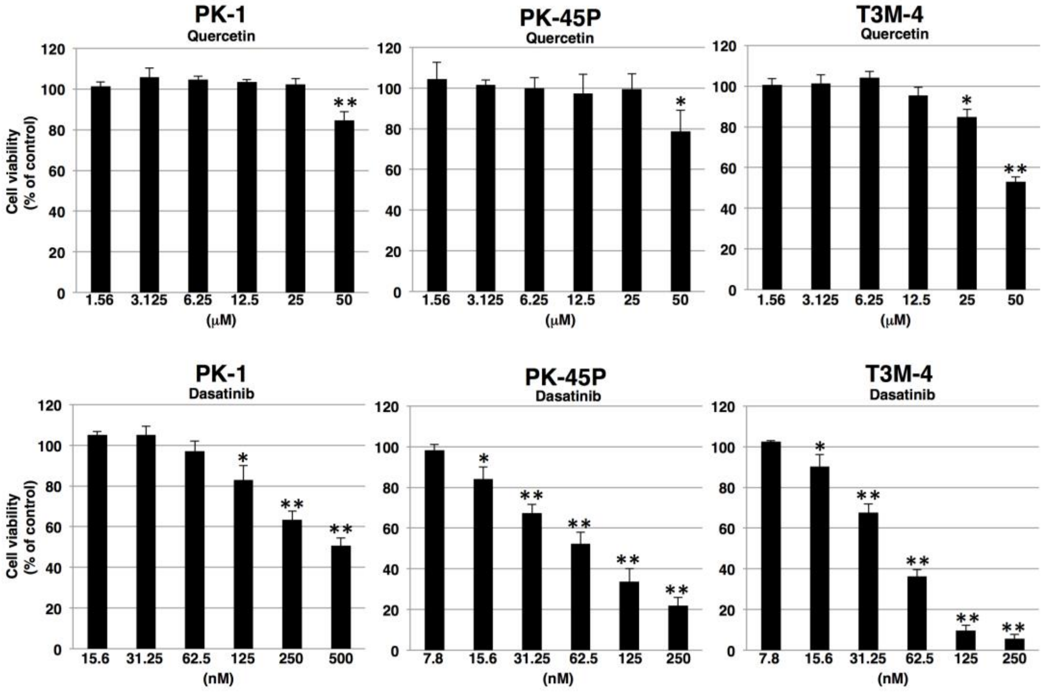

Figure S6. Dose-dependent effects of quercetin or dasatinib on the viability of PDAC cell lines. PK-1, PK-45P, and T3M-4 cells were incubated for 4 days with increasing concentrations of quercetin (1.56–50 μM) or dasatinib (7.8–500 nM). Cell viability was then determined by ATP assays. $*p < 0.05$, $**p < 0.01$ vs. 25 μM quercetin in PK-1 and PK-45P cells, 12.5 μM quercetin in T3M-4 cells, 62.5 nM dasatinib in PK-1 cells, 7.8 nM dasatinib in PK-45P and T3M-4 cells.

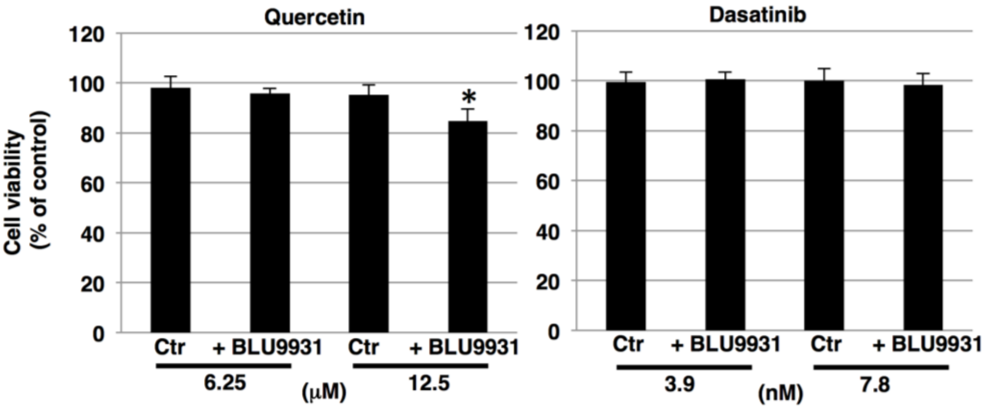

Figure S7. Effects of senolytic drug on BLU9931-induced senescent T3M-4 cells. T3M-4 cells were incubated for 7 days in the presence or absence of 2μM BLU9931. The cells were then incubated for 4 days with quercetin (6.25 or 12.5 μM) or dasatinib (3.9 or 7.8 nM), and cell viability was measured by ATP assays. $*p < 0.05$. Control (Ctr): Control cells were incubated with DMSO.

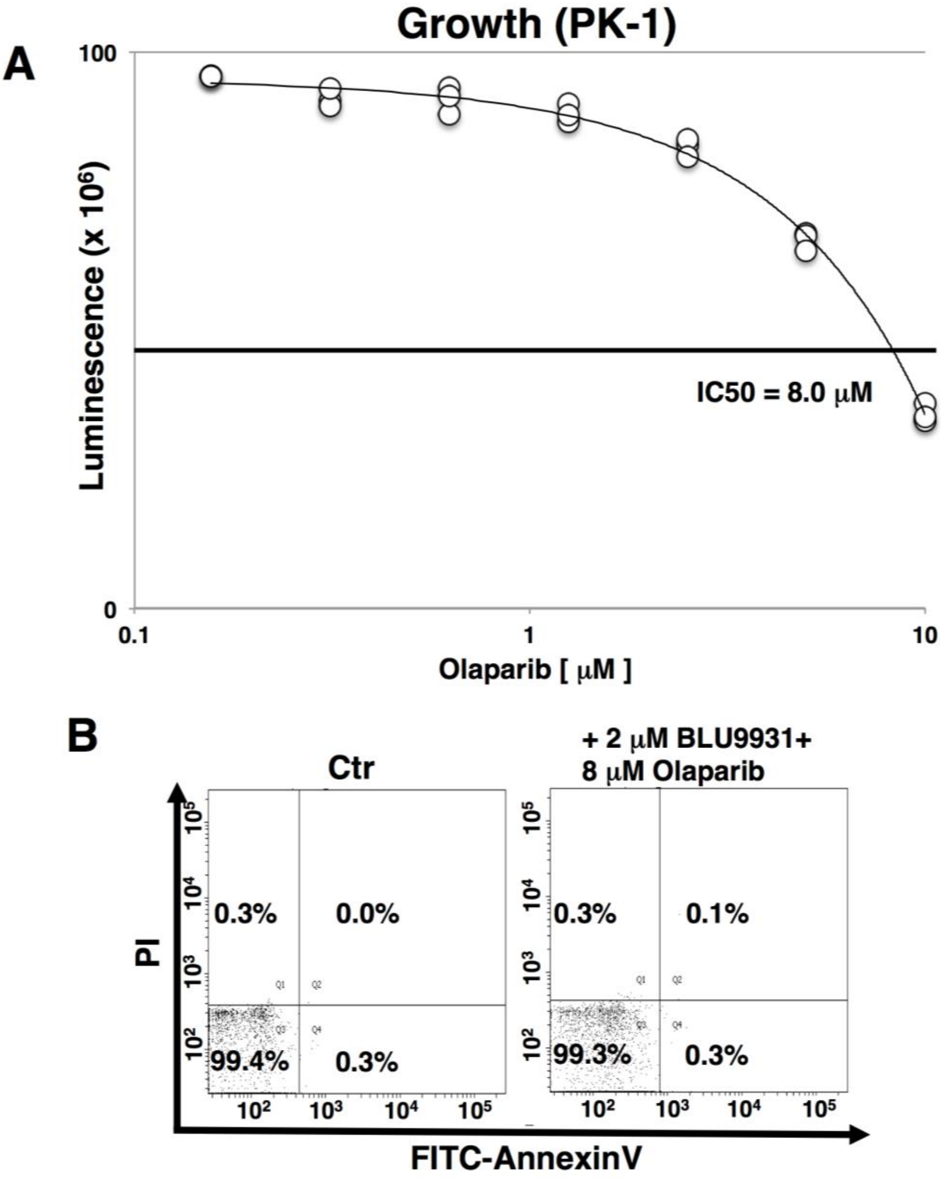

Figure S8. Effects of olaparib on the growth and viability of PK-1 cells. (A) PK-1 cells were incubated with increasing concentrations of olaparib (0.156–10 μM) for 3 days and growth rates were determined by ATP assays. (B) Quantification of apoptotic, necrotic, and live cells by flow cytometry in PK-1 cells incubated with or without 2μM BLU9931 + 8μM Olaparib for 3 days. Control (Ctr): Control cells were incubated with DMSO.

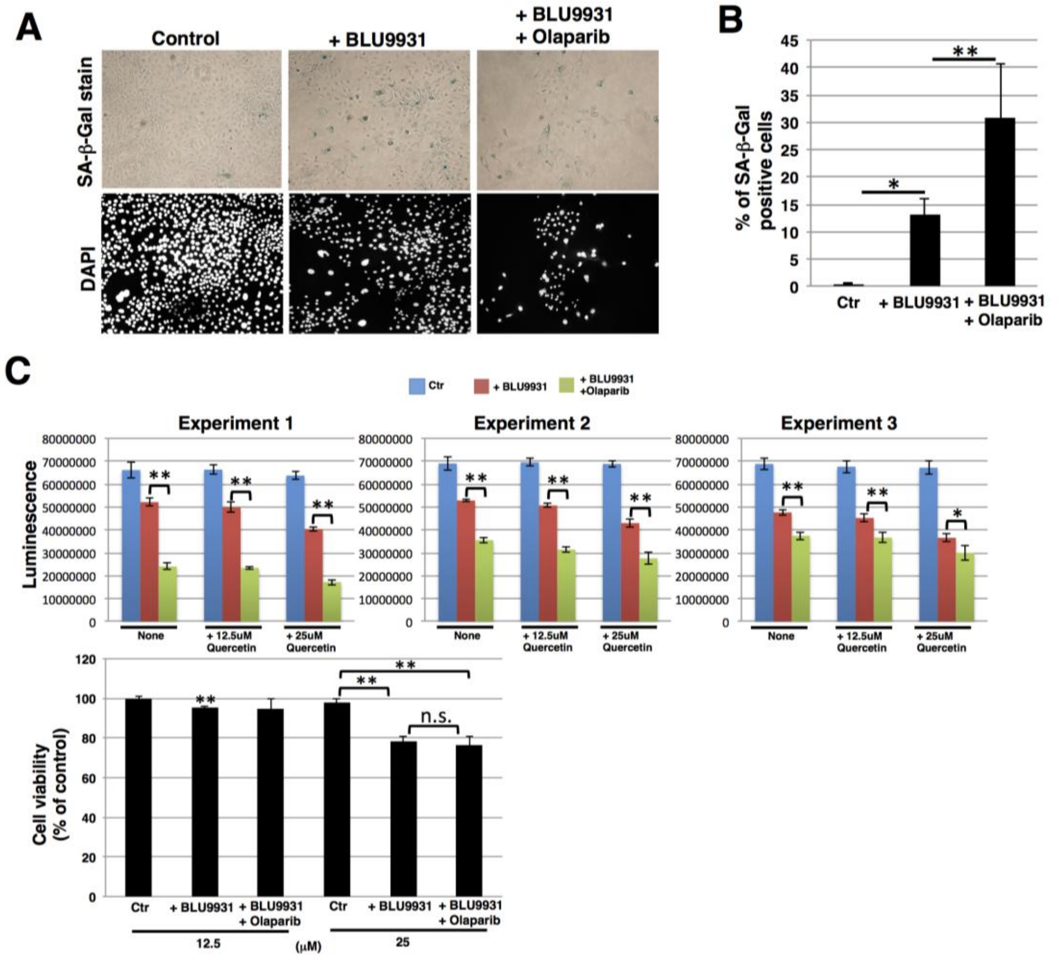

Figure S9. Effects of quercetin on SA-β-Gal staining and viability in PK-1 cells. (**A**) PK-1 cells that were incubated with or without 2μM BLU9931 ± 8μM Olaparib for 7 days were stained for SA-β-Gal activity. Representative images of staining for SA-β-Gal and DAPI are shown. (**B**) SA-β-Gal-positive cells in (**A**) were quantitated as a percentage of total cell numbers. *$p < 0.05$, **$p < 0.01$. (**C**) PK-1 cells were incubated for 7 days in the presence or absence of 2μM BLU9931 ± 8μM Olaparib. The cells were then incubated for 4 days with quercetin (6.25 or 12.5 μM), and cell viability was measured by ATP assays. Upper panels are three independent results of ATP assays. Lower panel show cell viability. *$p < 0.05$, **$p < 0.01$. n.s.: not significant. Control (Ctr): Control cells were incubated with DMSO.

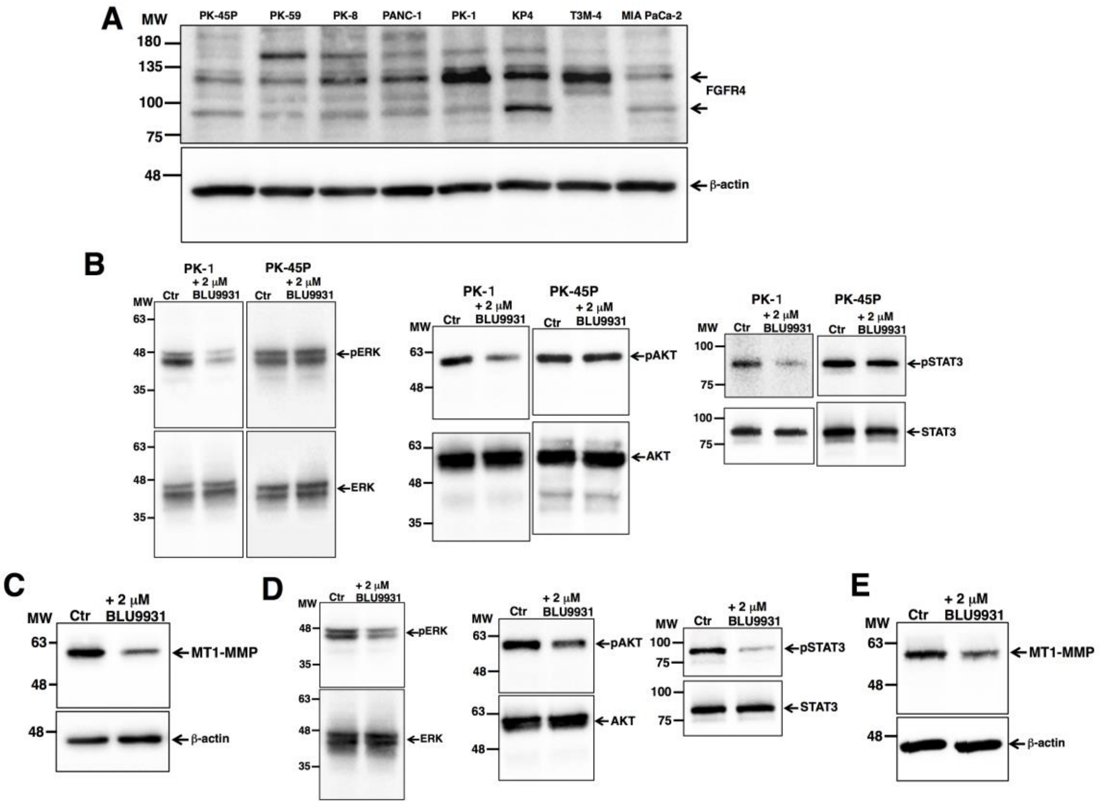

Figure S10. Western blots. Protein bands and molecular weight markers are shown for (**A**) Figure 1C, (**B**) Figure 3E, (**C**) Figure 5E, (**D**) Figure S3D, and (**E**) Figure S4B.

© 2020 by the authors. Licensee MDPI, Basel, Switzerland. This article is an open access article distributed under the terms and conditions of the Creative Commons Attribution (CC BY) license (http://creativecommons.org/licenses/by/4.0/).
